# Supplementary material for: HNF4α regulates sulfur amino acid metabolism and confers sensitivity to methionine restriction in liver cancer
Source: Nat Commun. 2020 Aug 7;11:3978. doi: 10.1038/s41467-020-17818-w (PMC7414133; doi:10.1038/s41467-020-17818-w)
Supplement: Supplementary file 1 — Supplementary Information [file 41467_2020_17818_MOESM1_ESM.pdf]

Supplementary Information

HNF4α regulates sulfur amino acid metabolism and confers sensitivity to methionine restriction in liver cancer

Xu et al.

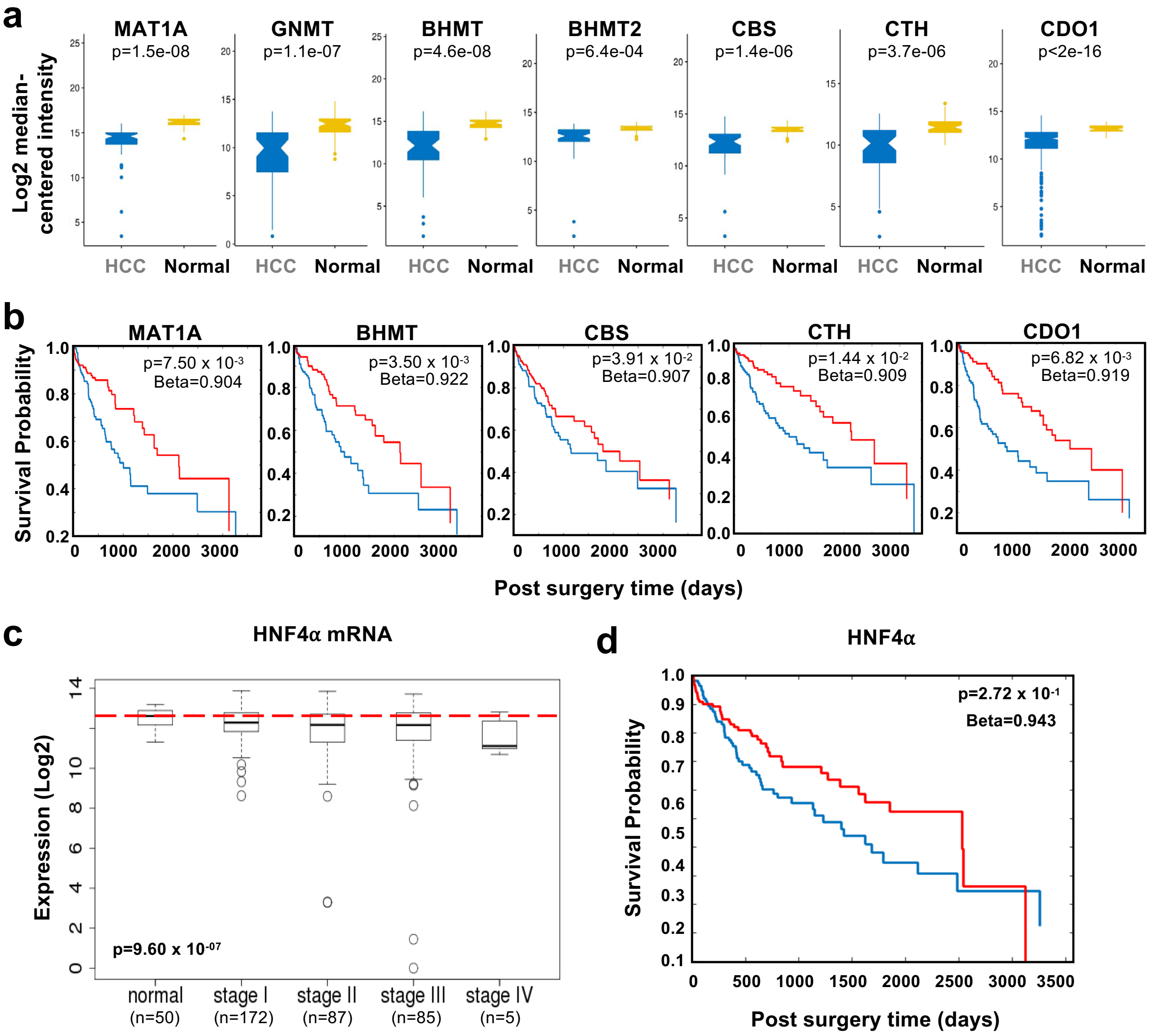

Supplementary Fig. 1. SAA metabolic enzymes and HNF4α are reduced and associated with patient survival in liver cancer patients.

a Expression of key SAA metabolic enzymes is reduced in HCC tumors. The processed TCGA RNA-seq gene expression data for n=50 normal and n=373 tumor LIHC samples were downloaded from TCGA data portal. **b** High expression levels of SAA metabolic enzymes are associated with increased survival in HCC patients (n=373). The coefficient estimate (beta value) and *p*-value using Cox proportional hazards regression were implemented in MATLAB, and the survival distribution was visualized with Kaplan-Meier survival curves. The Kaplan-Meier survival curves were generated using samples whose expression levels were among the top (red) and bottom (blue) 33% of expression values for corresponding genes. **c** HNF4α expression is reduced in HCC patients at different stages. The processed TCGA RNA-seq gene expression data for 50 normal and 373 tumor LIHC patients at different cancer stages (n=172 at Stage I, 87 at Stage II, 85 at Stage II, and 5 at Stage IV) were downloaded from TCGA data portal. **d** High expression of HNF4α is associated with increased survival of HCC patients. The Kaplan-Meier survival curves were generated using samples whose expression levels were among the top (red) and bottom (blue) 33% of expression values for HNF4α. For boxplots, the notch in (**a**) or the line in (**c**) is located at the median; the box length extends from the 1st quantile (Q1) to the 3rd quantile (Q3) which corresponds to the interquartile range (IQR). The maximum is located at Q3 + 1.5 x IQR whereas the minimum is located at Q1 – 1.5 x IQR. Points outside the maximum and the minimum were considered as outliers.

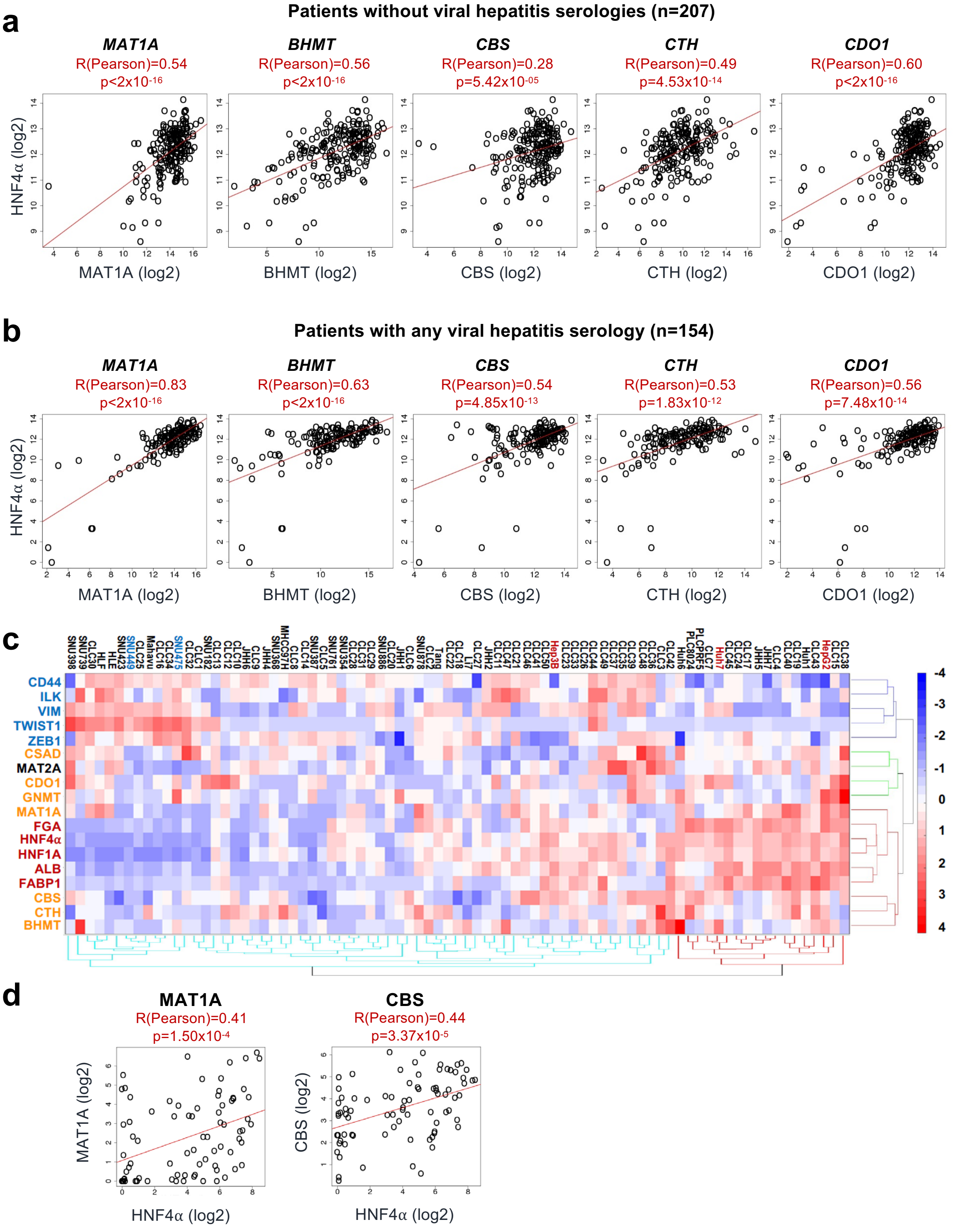

Supplementary Fig. 2. HNF4α and SAA metabolic enzymes are positively correlated in liver cancer patients and cells.

a Expression of key SAA metabolic enzymes is positively correlated with HNF4α expression in non-viral HCC patients (n=207). 207 patients without any viral hepatitis serologies were identified among 373 HCC patients in the TCGA LIHC dataset. The pair-wise Pearson correlation coefficient and the corresponding *p*-value between two genes among these patients were calculated using MATLAB. **b** The expression of key SAA metabolic enzymes is positively correlated with HNF4α expression in any viral HCC patients (n=154). There were 154 patients with any hepatitis viral serology (positive of hepatitis c antibody, hepatitis b surface antigen, hepatitis b surface antibody, hepatitis b core antibody, and/or hepatitis b DNA) in 373 HCC patients in the TCGA LIHC dataset. The pair-wise Pearson correlation coefficient and the corresponding *p*-value between two genes among these patients were calculated using MATLAB. **c** HNF4α is clustered together with key SAA metabolic enzymes in liver cancer cells. The mRNA levels of HNF4α-mediated liver functional genes (Red), mesenchymal markers (Blue), and SAA metabolic enzymes (Orange) from 81 liver cancer cells from LIMORE database [1] were clustered and represented by the heatmap using MATLAB as described in Methods. Please note that all liver cancer cells in this database were treated with a ROCK inhibitor Y-27632 and a transforming growth factor-β inhibitor A83-01 to inhibit mesenchymal cells while supporting epithelial cell growth [1]. **d** The expression of MAT1A and CBS is positively correlated with HNF4α expression in liver cancer cells from LIMORE database (n=81 liver cancer cell lines).

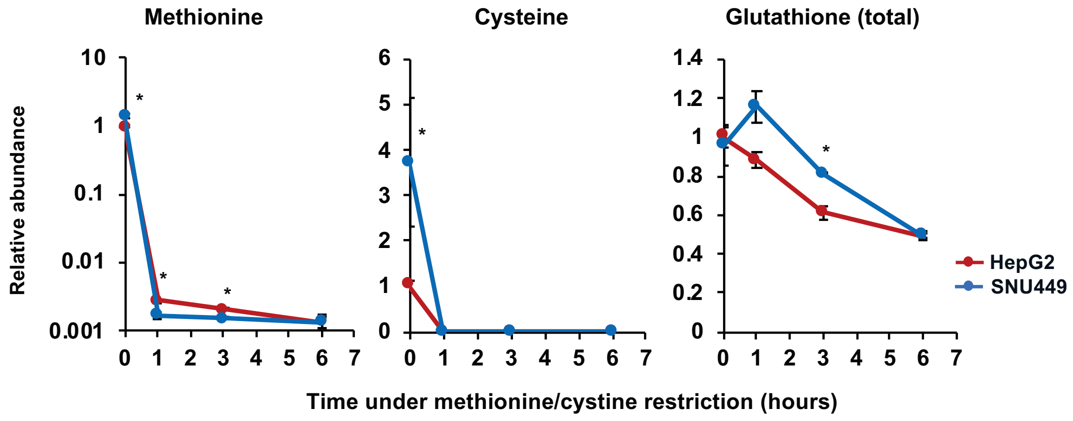

Supplementary Fig. 3. Methionine/cysteine restriction quickly depletes cellular methionine and cysteine.

HepG2 and SNU449 cells were cultured in methionine/cysteine restricted medium for indicated times, and the intracellular methionine, cysteine, and glutathione levels were quantified by LC-MS (n=3 replicates per group, values are expressed as mean ± s.e.m., two-tailed unpaired Student's t-test, *p<0.05).

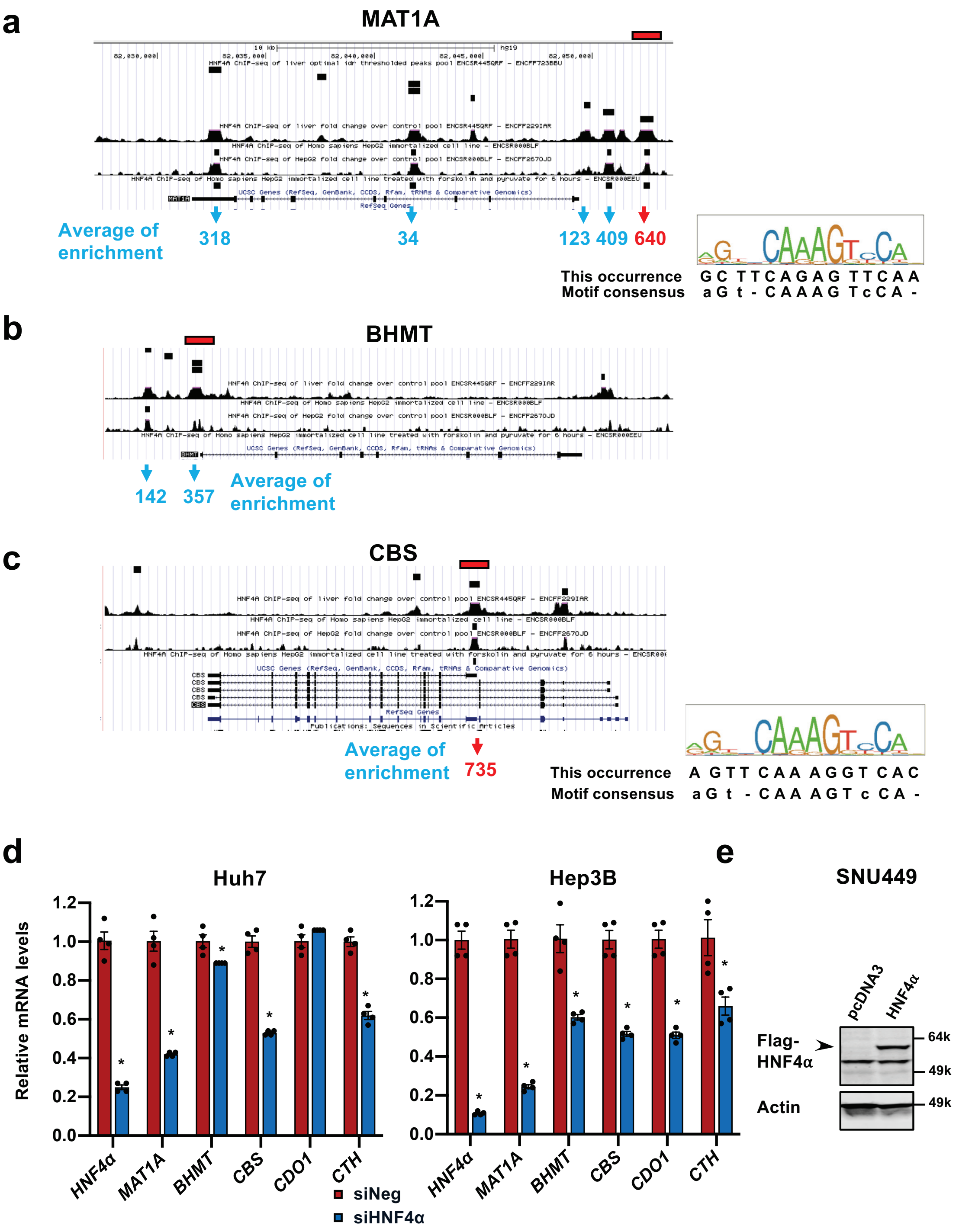

Supplementary Fig. 4. HNF4α regulates the transcription of key SAA metabolic genes.

a-c Key SAA metabolic enzymes have HNF4α binding sites on their promoters. Publicly available data for the HNF4α ChIP-seq from human liver or HepG2 cells from the ENCODE project [2] were displayed in UCSC genome browser for MAT1A (**a**), BHMT (**b**), and CBS (**c**) genes, and the enrichment scores and consensus HNF4α binding sites are shown for peaks indicated by the red arrows. Blue arrows indicate additional ChIPseq peaks. Red bars: promoter regions cloned and mutated in the luciferase assay. **d** Knockdown of HNF4α reduces the mRNA levels of key SAA metabolic enzymes in Huh7 and HepB3 cells. Huh7 and HepB3 cells were transfected with control siRNA (siNeg) or siRNAs against HNF4α (siHNF4α) for 48 hours. mRNA levels of indicated SAA genes were analyzed by qPCR (n=4 replicates per group). **e** Overexpression of HNF4α in SNU449 cells for the luciferase reporter assay (relative to Fig. 4**d**). HNF4α-negative SNU449 cells were transfected with the control vector (pcDNA3) or a construct expressing HNF4α, and the total cell lysates were immuno-blotted with an anti-HNF4α antibody (representative blots were shown from at least three independent experiments). For bar graphs in (**d**), values are expressed as mean ± s.e.m., two-tailed, unpaired Student's t-test, $*p<0.05$.

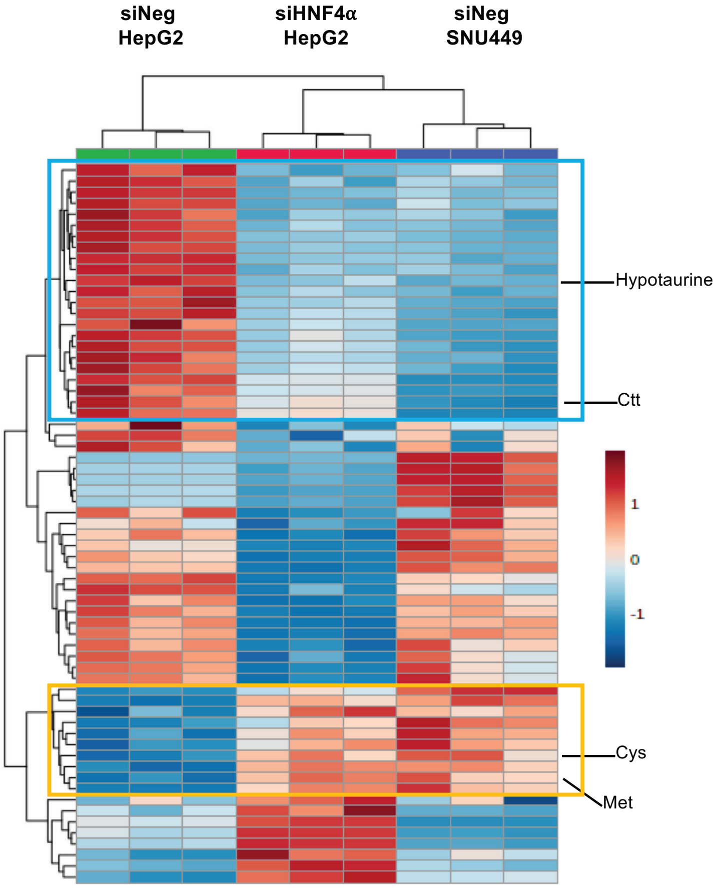

Supplementary Fig. 5. HNF4α deficiency impairs SAA metabolism.
HNF4α-depleted HepG2 cells and HNF4α-negative SNU449 cells are metabolically clustered together. A cluster analysis was performed using all significantly ($p<0.05$, $|FC|>1.5$) changed metabolites in siHNF4α vs siNeg HepG2 cells. Blue or orange boxes highlight two major metabolite clusters that are altered in both HNF4α-depleted HepG2 cells and HNF4α-negative SNU449 cells.

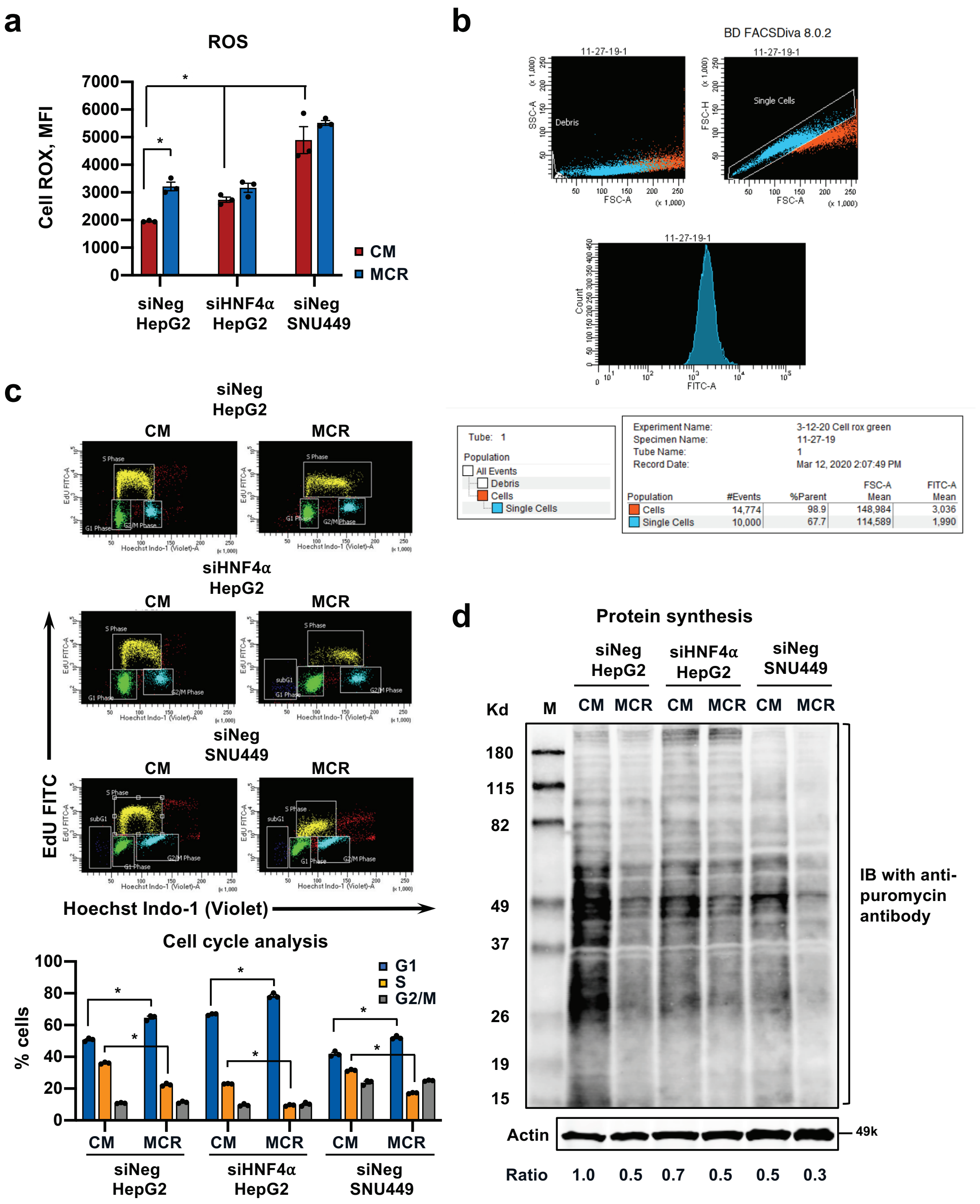

Supplementary Fig. 6. HNF4α deficiency reduces the sensitivity to SAA restriction.
a HNF4α-depleted HepG2 cells and HNF4α-negative SNU449 cells have increased ROS levels and are not sensitive to further methionine/cystine restriction-induced ROS production (n=3 replicates per group). Indicated cells were cultured in CM and MCR medium for 18 hours. Cells were incubated with CellROX Green for 30 minutes and cellular ROS levels were analyzed by flow cytometry. **b** Gating strategy for defining single-cell populations and representative histogram of CellROX Green fluorescence. **c** Methionine/cystine restriction induces G1 arrest independently of the cellular HNF4α status (n=3 replicates per group). Cells were cultured in CM and MCR medium for 18 hours followed by one-hour incubation with EdU. Cell cycle was analyzed with Click-iT EdU Flow Cytometry Assay Kit. Single-cells population gating was performed as in (b). Gating of the cell cycle phases is shown in the top panel and quantification of percentages of cells in the different cell cycle phases is presented in the bottom panel. **d** Methionine/cystine restriction suppresses protein synthesis independently of the cellular HNF4α status. Indicated cells were cultured in complete medium (CM) and methionine/cystine restricted medium (MCR) medium for 18 hours. 10 μM puromycin were then added to the medium for 10 minutes, and cells were washed with PBS and protein lysates were immuno-blotted with anti-puromycin and anti-actin antibodies. The intensity ratios of puromycin-labeled peptides vs actin control were calculated using Fiji software. For bar graphs in (**a**, **c**), values are expressed as mean ± s.e.m., two-tailed, unpaired Student's t-test, *p<0.05.

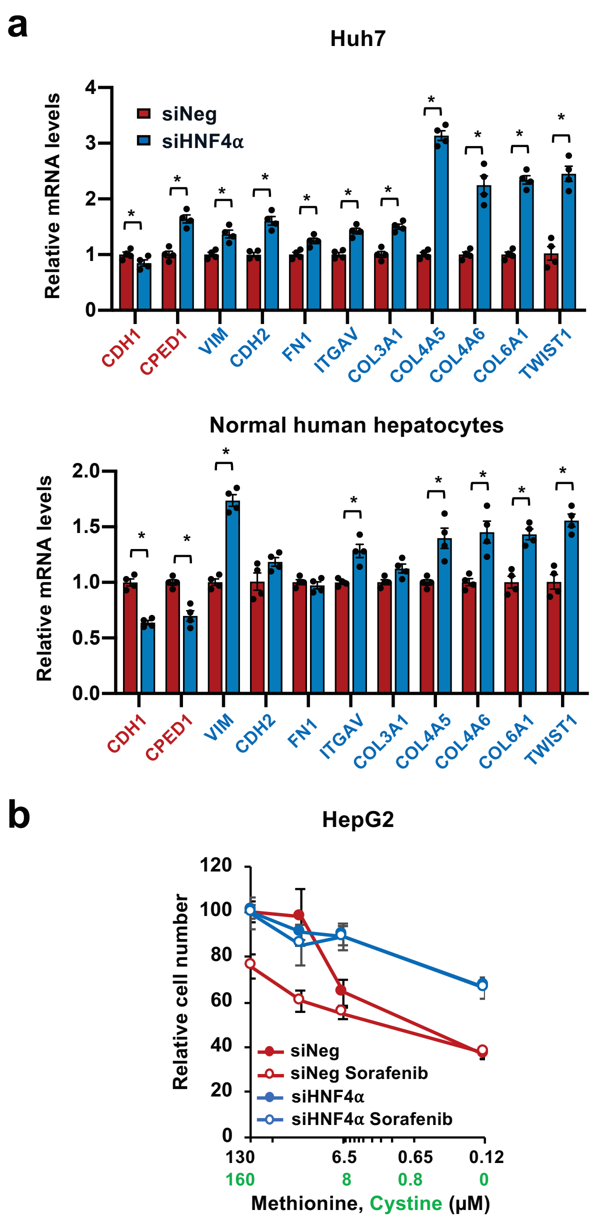

Supplementary Fig. 7. HNF4α depletion increases EMT and resistance to methionine restriction in HNF4α-positive liver cancer cells

a Knocking down HNF4α represses the expression of epithelial markers while inducing mesenchymal markers. The expression of indicated epithelial (Red) and mesenchymal (Blue) markers were analyzed in control (siNeg) and HNF4α knockdown (siHNF4α) Huh7 and normal human hepatocytes by qPCR (n=4 replicates per group). **b** HNF4α-depleted HepG2 cells are resistant to cell death induced by methionine/cystine restriction and sorafenib. Control (siNeg) and siHNF4α HepG2 cells were cultured in medium containing indicated amount of methionine/cystine, together with or without 5 μM sorafenib for 24 hours. The relative number of surviving cells was measured by the WST-1 assay (n=3 replicates per group). For all graphs, values are expressed as mean ± s.e.m., two-tailed, unpaired Student's t-test, *p<0.05.

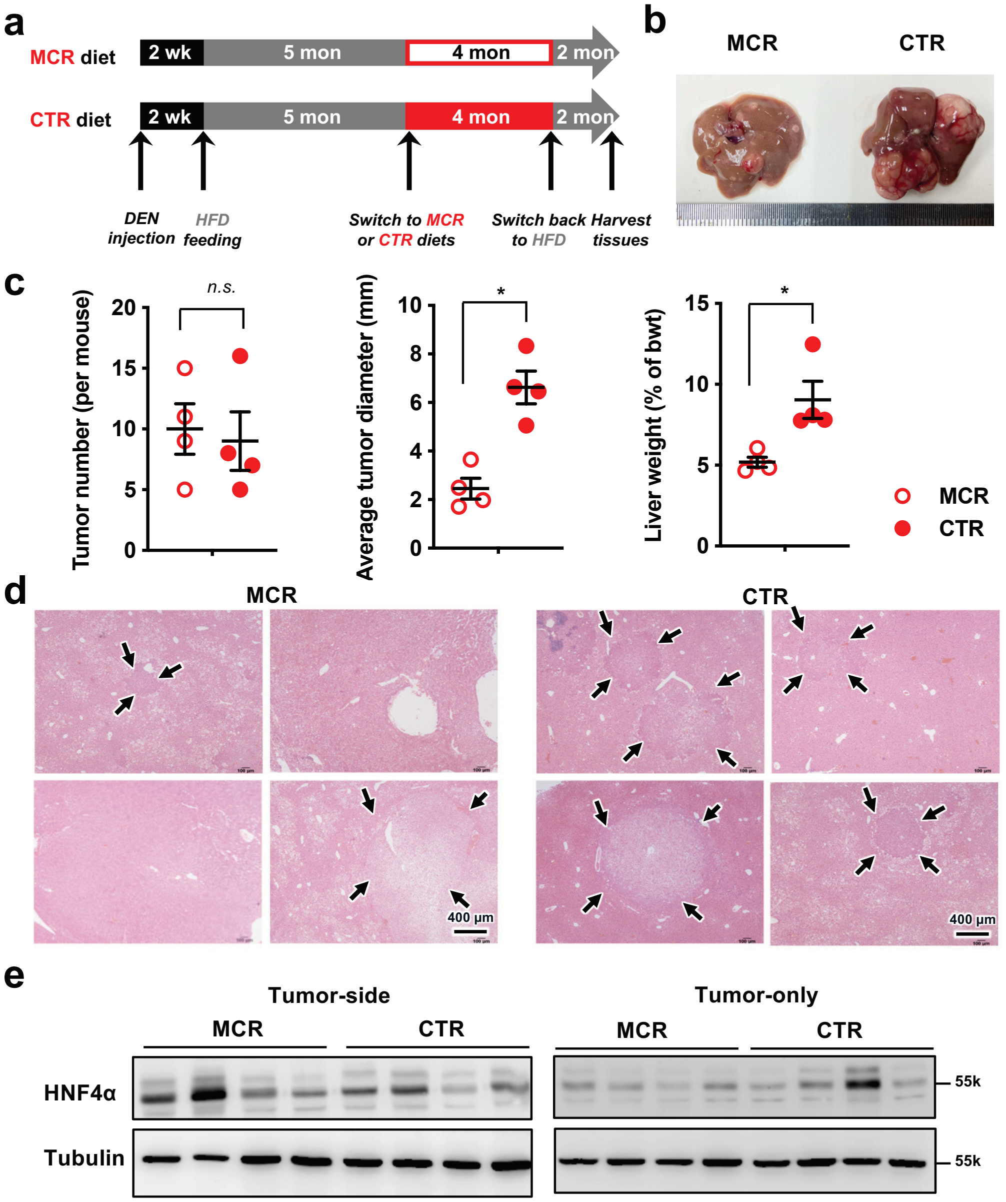

Supplementary Fig. 8. MCR diet feeding suppresses liver tumorigenesis *in vivo*.
a Experimental scheme of methionine/cystine restriction (MCR) in DEN/HFD-induced liver cancer formation. **b-d** Four-month feeding of MCR diet suppresses liver cancer growth. C57BL/6 mice under the DEN/HFD liver cancer model were fed with a control diet (CTR) or methionine restricted diet (MCR) for four months as indicated in **a**. The liver images (**b**), liver tumor number, tumor size, and liver weight (**c**), and liver histology (**d**) were analyzed at the end of the procedure (n=4 mice in CTR and 4 mice in MCR). H&E images in (**d**) are from 4 individual animals; arrows: tumors. **e** The expression of HNF4α in final tumor-side tissues (left) and tumors (right) were analyzed by immunoblotting (n=4 mice in CTR and 4 mice in MCR). For dot plots in (**c**), dots depict individual mice, values are expressed as mean ± s.e.m., two-tailed, unpaired Student's t-test, *p<0.05.

a

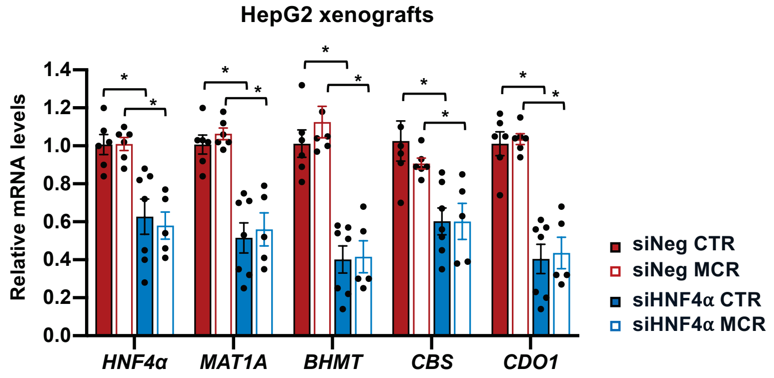

b

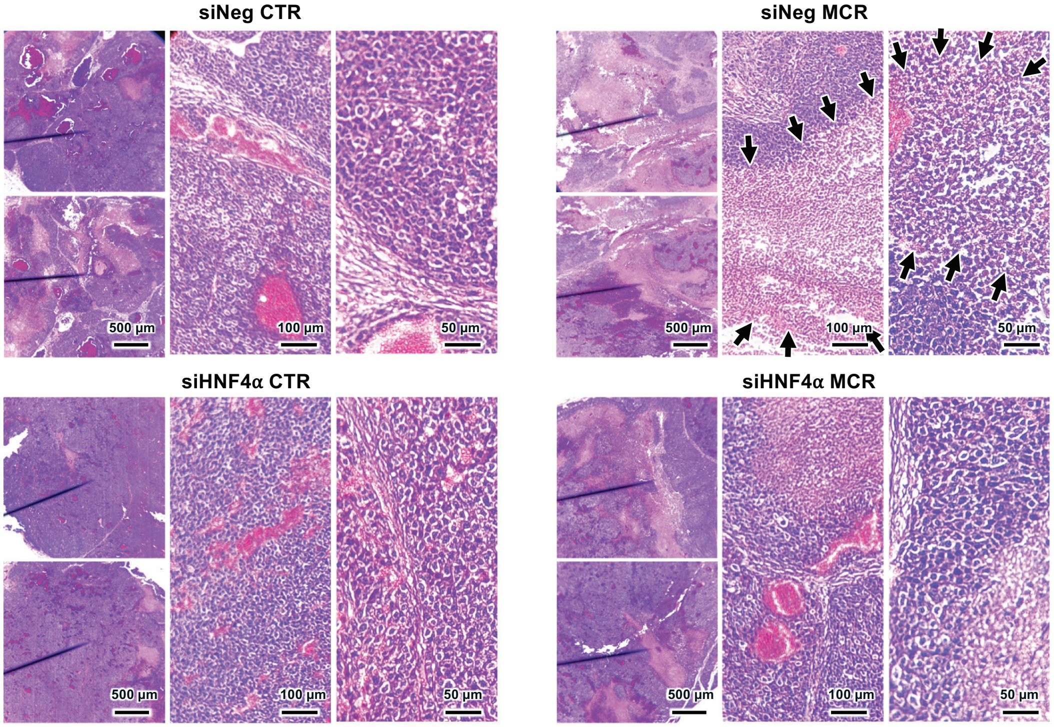

Supplementary Fig. 9. HNF4α depletion increases resistance to methionine restriction in HNF4α-positive liver cancer cells *in vivo*.

a The mRNA levels of HNF4α and key SAA metabolic enzymes in HepG2 xenografted tumors. Mice bearing control (siNeg) and siHNF4α HepG2 xenografted tumors were fed with a control diet (CTR) or methionine restricted diet (MCR) as described in Methods, and the expression of indicated genes was analyzed in final dissected tumors by qPCR (n=6 tumors in siNeg CTR, 6 tumors in Neg MCR, 7 tumors in siHNF4α CTR, and 5 tumors in siHNF4α MCR). **b** Histology of xenograft tumors. Representative H&E images of control (siNeg) and siHNF4α HepG2 xenografted tumors fed with a control diet (CTR) or methionine restricted diet (MCR) as in (**a**). Note that MCR-fed siNeg tumors displayed increased damage and death (arrows). For the bar graph in (**a**), dots depict individual mice, values are expressed as mean ± s.e.m., two-tailed, unpaired, non-parametric Mann-Whitney test, *p<0.05.

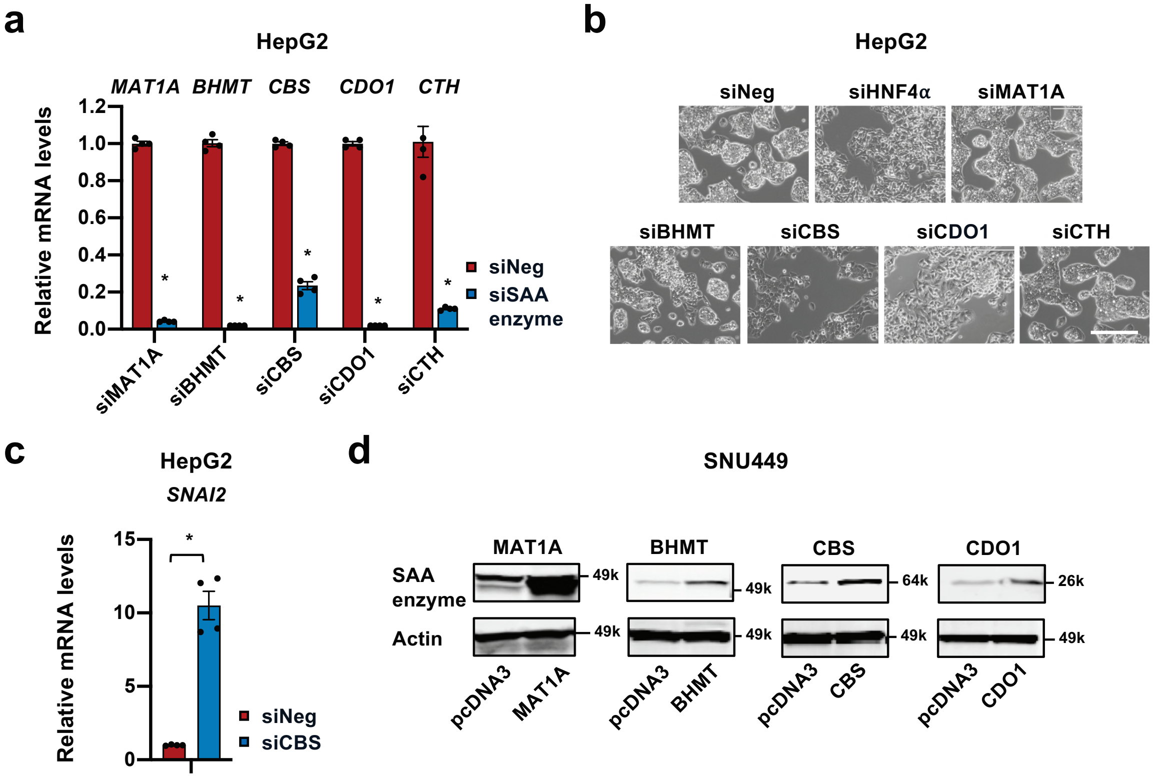

Supplementary Fig. 10. Manipulation of individual SAA metabolism enzyme affects EMT.

a The expression of SAA enzymes after knockdown of individual SAA enzyme in HepG2 cells. Control (siNeg) HepG2 cells, and HepG2 cells with knockdown of individual SAA enzyme were analyzed for expression of the indicated targeted gene by qPCR (n=4 replicates per group). **b** Knockdown of individual SAA enzyme induces morphological alterations in HepG2 cells. Representative images were shown from at least three independent experiments. Bar, 400 μm. **c** Knocking down CBS induces SNAI2 expression in HepG2 cells (n=4 replicates per group). **d** Overexpression of individual SAA enzyme in HNF4α-negative SNU449 cells. The expression of indicated SAA enzymes was analyzed by immuno-blotting (representative blots were shown from at least three independent experiments). For bar graphs in (**a**, **c**), values are expressed as mean ± s.e.m., two-tailed, unpaired Student's t-test, *p<0.05.

Supplementary Table 1 Metabolites that are significantly altered in SNU449 cells vs HepG2 cells

Compound name	HMDB	PubChem	KEGG	Relative abundance		* p value
				HepG2	SUN449	
IMP	HMDB00175	8582	C00130	1	0.00	0.0247
5-Phospho-beta-D-ribosylamine	HMDB01128	439905	C03090	1	0.00	0.0162
N2-Formyl-N1-(5-phospho-D-ribosyl)glycinamide	METPA0491	NA	C04376	1	0.01	0.0053
C9H13N5O3(4)	NA	NA	NA	1	0.01	0.0264
N-Carbamoyl-L-aspartate	HMDB00828	93072	C00438	1	0.03	0.0023
N-phosphocreatinate	HMDB01511	587	C02305	1	0.05	0.0004
ribose-phosphate	HMDB01548	3417	C00117	1	0.07	0.0027
Sedoheptulose 7-phosphate	METPA0585	NA	C05382	1	0.08	0.0070
dUDP	HMDB01000	145729	C01346	1	0.08	0.0013
O-Phospho-L-serine	HMDB01721	106	C01005	1	0.11	0.0150
S-Adenosyl-L-homocysteine	HMDB00939	439155	C00021	1	0.12	0.0169
6-Phosphogluconic acid	HMDB01316	91493	C00345	1	0.12	0.0235
dUMP	HMDB01409	65063	C00365	1	0.12	0.0004
Glycerol 3-phosphate	HMDB00126	439162	C00093	1	0.12	0.0012
3-methyl-2-oxopentanoate/4-methyl-2-oxopentanoate(ketoleu	HMDB00491	47	C03465	1	0.13	0.0018
D-Erythrose 4-phosphate	HMDB01321	122357	C00279	1	0.13	0.0040
(S)-dihydroorotate	HMDB03349	439216	C00337	1	0.14	0.0000
L-cysteinylglycine	HMDB00078	439498	C01419	1	0.16	0.0085
decenoyl carnitine	NA	NA	NA	1	0.17	0.0065
S-adenosyl-L-methionine	HMDB01185	16757548	C00019	1	0.18	0.0116
fructoselysine 3-phosphate	NA	NA	NA	1	0.18	0.0012
G6P/F6P	HMDB01401	5958	C00092	1	0.19	0.0044
decanoyl carnitine	HMDB06210	53477803		1	0.19	0.0002
Deoxyinosine	HMDB00071	65058	C05512	1	0.19	0.0008
Cystathionine	HMDB00099	439258	C02291	1	0.20	0.0072
glutathione	HMDB00125	124886	C00051	1	0.21	0.0130
C27H46O4(3)				1	0.23	0.0133
D-Glucuronate 1-phosphate	HMDB03976	440650	C05385	1	0.23	0.0009
adenosine	HMDB00050	60961	C00212	1	0.24	0.0024
UDP-D-glucuronate	HMDB00935	17473	C00167	1	0.25	0.0013
ADP-mannose/GDP-L-fucose/ADP alpha-D-glucoside	HMDB06369	440940	C06192	1	0.26	0.0014
L-xylonate/L-lyxonate	HMDB60256	6971043	C05411	1	0.27	0.0025
Hypotaurine	HMDB00965	107812	C00519	1	0.29	0.0004
N(omega)-(L-Arginino)succinate	HMDB00052	16950	C03406	1	0.29	0.0062
2-oxoglutarate(2-)	HMDB00208	51	C00026	1	0.30	0.0107
11'-Carboxy-alpha-chromanol	HMDB12515	53481451		1	0.36	0.0038
trans-caffeate/3-(4-hydroxyphenyl)pyruvate	HMDB03501	689043	C01197	1	0.38	0.0057
octanoyl carnitine/L-Octanoylcarnitine	HMDB00791	11953814	C02838	1	0.39	0.0020
(R)-carnitine	HMDB00062	2724480	C15025	1	0.40	0.0018
acetylcholine/4-(trimethylammonio)butanoate	HMDB00895	187	C01996	1	0.40	0.0063
lauroyl carnitine	HMDB02250	168381		1	0.42	0.0010
pseudoecgonine/ecgonine	HMDB06348	443845	C12449	1	0.43	0.0002
O-acetylcarnitine	HMDB00201	1	C02571	1	0.46	0.0017
gulonate	HMDB03290	152304	C00800	1	0.46	0.0003
guanosine	HMDB00133	6802	C00387	1	0.46	0.0008
L-asparagine	HMDB00168	6267	C00152	1	0.46	0.0092
Ureidopropionic acid/Glycyl-glycine	HMDB00026	111	C02642	1	0.46	0.0093
3-Hydroxybutyrylcarnitine	HMDB13127	53481617Ê	NA	1	0.50	0.0006
UDP-N-acetyl-alpha-D-glucosamine/UDP-N-acetyl-alpha-D	HMDB00290	445675	C00043	1	0.53	0.0019
octenoyl carnitine	NA	NA	NA	1	0.53	0.0015
L-arabinitol/xylitol/D-ribitol	NA	NA	NA	1	0.54	0.0018
C27H44O3(5)	NA	NA	NA	1	0.59	0.0087
choline phosphate	HMDB01565	1014	C00588	1	0.59	0.0042

aspartate	HMDB00191	5960	C00049	1	0.60	0.0049
CMP-N-trimethyl-2-aminoethylphosphonate	HMDB60072	440755	C05674	1	0.61	0.0020
fumarate	HMDB00134	444972	C00122	1	0.62	0.0071
CDP-ethanolamine	HMDB01564	123727	C00570	1	0.62	0.0344
cis-aconitate	HMDB00072	643757	C00417	1	0.62	0.0305
CDPcholine	HMDB01413	13804	C00307	1	0.63	0.0105
citrate/isocitrate	HMDB00094	311	C00158	1	0.63	0.0310
CMP	HMDB00095	6131	C00055	1	0.64	0.0058
pyruvate	HMDB00243	1060	C00022	1	0.65	0.0133
malate(2-)	HMDB00156	222656	C00149	1	0.65	0.0162
N-acetyl-seryl-aspartate	NA	NA	NA	1	0.66	0.0118
C18H32O4(3)	NA	NA	NA	1	1.50	0.0062
(R)-mevalonate	HMDB59629	439230	C00408	1	1.51	0.0188
NN-dimethylglycine	HMDB00092	673	C01026	1	1.52	0.0290
3(S)10(R)-OH-octadeca-6-trans-412-cis-trienoate	NA	NA	NA	1	1.53	0.0063
Hexadecanedioic acid	HMDB00672	10459	C19615	1	1.53	0.0091
sphingosine/(2S)-1-hydroxy-3-oxooctadecan-2-aminium	HMDB00252	5353955	C00319	1	1.54	0.0169
arginine	HMDB00517	6322	C00062	1	1.55	0.0345
creatinine	HMDB00562	588	C00791	1	1.55	0.0100
Pyridoxine/Norepinephrine-1	HMDB00239	1054	C00314	1	1.56	0.0366
beta-hydroxy-beta-methylbutyrate	HMDB00754	69362		1	1.56	0.0146
L-tyrosine	HMDB00158	6057	C00082	1	1.57	0.0265
lactate	HMDB00190	107689	C00186	1	1.57	0.0002
adrenochrome/N-benzoylglycinate	HMDB12884	5898		1	1.57	0.0443
benzoate	HMDB01870	243	C00180	1	1.58	0.0329
C9H8O3(3)	NA	NA	NA	1	1.58	0.0247
coumarin	HMDB01218	323	C05851	1	1.59	0.0254
D-Glucosamine	HMDB01514	439213	C00329	1	1.59	0.0092
phenylacetaldehyde	HMDB06236	998	C00601	1	1.62	0.0421
stearate	HMDB00827	5281	C01530	1	1.62	0.0186
5-oxoproline/L-1-Pyrroline-3-hydroxy-5-carboxylate	HMDB00267	4992	C01879	1	1.62	0.0406
Nicotinamide	HMDB01406	936	C00153	1	1.63	0.0477
2-Hydroxybutyric acid	HMDB00008	11266	C05984	1	1.64	0.0098
Hypoxanthine	HMDB00157	790	C00262	1	1.64	0.0236
L-methionine	HMDB00696	6137	C00073	1	1.64	0.0153
L-4-hydroxyglutamate semialdehyde	HMDB06556	8224	C05938	1	1.65	0.0012
margarate	HMDB02259	10465		1	1.66	0.0105
C20H30O4(18)	NA	NA	NA	1	1.67	0.0277
C4H6O3(3)				1	1.67	0.0146
omega hydroxy hexadecanoate (n-C16:0)	HMDB06294	7058075		1	1.67	0.0173
L-tryptophan	HMDB00929	6305	C00078	1	1.68	0.0452
9,10-hydroxyoctadec-12(Z)-enoate/12,13-hydroxyoctadec-9(Z	HMDB04704	9966640	C14828	1	1.68	0.0001
Xanthine	HMDB00292	1188	C00385	1	1.68	0.0448
L-Alanyl-L-leucine	HMDB28691	NA	NA	1	1.69	0.0230
indole-3-acetate/(5-hydroxyindol-3-yl)acetaldehyde	HMDB00197	802	C00954	1	1.69	0.0272
alpha-D-glucose	HMDB03345	79025	C00267	1	1.69	0.0075
4-pyridoxate	HMDB00017	6723	C00847	1	1.70	0.0177
2-keto-4-methylthiobutyrate	HMDB01553	473	C01180	1	1.70	0.0246
N-formylanthranilate/noradrenochrome	HMDB04089	101399	C05653	1	1.73	0.0053
succinyl carnitine	NA	NA	NA	1	1.73	0.0120
carnosine	HMDB00033	439224	C00386	1	1.73	0.0303
CDP	HMDB01546	6132	C00112	1	1.73	0.0075
L-phenylalanine	HMDB00159	6140	C00079	1	1.75	0.0044
succinate(2-)	HMDB00254	1110	C00042	1	1.75	0.0178
Methionine sulfoxide	HMDB02005	847		1	1.76	0.0037
L-erythrulose	HMDB06293	5460032	C02045	1	1.78	0.0110
C5H10O5(6)	NA	NA	NA	1	1.78	0.0122
citr_L[e]	NA	NA	NA	1	1.80	0.0074

gama-L-glutamyl-L-alpha-aminobutyrate	NA	NA	NA	1	1.80	0.0382
2-methylglutaconic acid	HMDB02266	6368126		1	1.81	0.0101
4-Hydroxyphenylacetate/2-Hydroxyphenylacetate/3,4-Dihydro	HMDB00020	127	C00642	1	1.81	0.0129
beta-carboline	HMDB12897	64961	C20157	1	1.83	0.0018
Biotin	HMDB00030	171548	C00120	1	1.86	0.0119
L-isoleucine/L-leucine	HMDB00172	6306	C00407	1	1.86	0.0069
C6H11NO3(4)	NA	NA	NA	1	1.86	0.0005
oleic acid/elaidate/trans-vaccenate	HMDB00207	445639	C00712	1	1.87	0.0484
L-histidine	HMDB00177	6274	C00135	1	1.87	0.0260
naphthalene	HMDB29751	931	C00829	1	1.91	0.0264
Deoxyuridine	HMDB00012	13712	C00526	1	1.93	0.0129
myo-inositol	HMDB00211	NA	C00137	1	1.96	0.0139
C6H12O6(5)	NA	NA	NA	1	1.97	0.0150
Valine	HMDB00883	6287	C00183	1	1.98	0.0015
C20H32O4(21)	NA	NA	NA	1	1.99	0.0205
(R)-S-lactoylglutathionate	HMDB01066	440018	C03451	1	2.02	0.0065
2-Ketobutyric acid	HMDB00005	58	C00109	1	2.02	0.0008
Trimethylamine N-oxide	HMDB00925	1145	C01104	1	2.04	0.0015
acetoacetate	HMDB00060	96	C00164	1	2.05	0.0007
5-Hydroxyindoleacetate	HMDB00763	1826	C05635	1	2.05	0.0009
N-acetylneuraminic acid	HMDB00230	445063	C19910	1	2.05	0.0081
L-kynurenine/Formyl-5-hydroxykynurenamine	HMDB00684	161166	C00328	1	2.06	0.0003
cytidine	HMDB00089	6175	C00475	1	2.07	0.0043
CTP	HMDB00082	6176	C00063	1	2.12	0.0046
phytanic acid/arachidic acid	HMDB00801	26840	C01607	1	2.12	0.0032
cholesterol sulfate	HMDB00653	65076	C18043	1	2.16	0.0149
methyl indole-3-acetate	HMDB29738	74706		1	2.16	0.0009
C20H30O3(6)	NA	NA	NA	1	2.20	0.0323
allantoate	HMDB01209	203	C00499	1	2.23	0.0300
linoleic acid/linoelaidic acid (all trans C18:2)/octadecadienoat	HMDB00673	5280450	C01595	1	2.27	0.0075
choline	HMDB00097	305	C00114	1	2.28	0.0315
Glycylleucine	HMDB00759	92843	C02155	1	2.35	0.0149
beta-D-Fructose 1,6-bisphosphate	HMDB60444	10267	C05378	1	2.36	0.0120
L-Iditol/D-glucitol/galactitol	NA	NA	NA	1	2.38	0.0046
tetradecanoyl carnitine(myristoyl carnitine)	HMDB05066	53477791		1	2.41	0.0112
quinolinate	HMDB00232	1066	C03722	1	2.43	0.0005
docosahexaenoate(DHA)	HMDB02183	445580	C06429	1	2.44	0.0118
8,11,14-Eicosatrienoic acid (DGLA)	HMDB02925	5280581	C03242	1	2.46	0.0061
N-Methylethanolamine phosphate	NA	NA	C01210	1	2.59	0.0036
L-fucose/L-Fuculose	HMDB00174	17106	C01019	1	2.62	0.0019
GDP-D-mannose	HMDB01163	18396	C00096	1	2.68	0.0452
N-acetyl-L-glutamate	HMDB01138	185	C00624	1	2.73	0.0037
C20H30O2(4)	NA	NA	NA	1	2.79	0.0048
sn-Glycero-3-phosphocholine	HMDB00086	71920	C00670	1	2.88	0.0040
C20H32O3(12)	NA	NA	NA	1	2.91	0.0479
trans-Hexadec-2-enoyl carnitine	HMDB06317	53477817		1	2.92	0.0057
L-cysteine	HMDB00574	5862	C00097	1	2.95	0.0180
acetylcarnosine	HMDB12881	10221026		1	3.12	0.0069
N(2)-acetyl-L-ornithine	HMDB03357	439232	C00437	1	3.25	0.0005
alpha-linolenyl carnitine/gamma-linolenyl carnitine	HMDB06319	53477821		1	3.35	0.0085
arachidonic acid	HMDB01043	444899	C00219	1	3.51	0.0023
5-guanidino-2-oxopentanoic acid	HMDB04225	558	C03771	1	3.69	0.0054
Galactosylglycerol	HMDB06790	656504	C05401	1	4.05	0.0075
clupanodonic acid/docosa-4,7,10,13,16-pentaenoic acid	HMDB06528	5497182	C16513	1	4.44	0.0039
pendtadenoyl carnitine	NA	NA	NA	1	4.78	0.0043
Linoleyl carnitine/Linoelaidyl carnitine	HMDB06469	6450015		1	6.17	0.0042
Elaidic carnitine/Vaccenyl carnitine	HMDB06464	53477837		1	7.09	0.0028
L-palmitoylcarnitine	HMDB00222	11953816	C02990	1	8.08	0.0015

dIMP	HMDB06555	91531	C06196	1	10.93	0.0342
heptadecanoyl carnitine	HMDB06210	53477803		1	13.21	0.0013
stearoylcarnitine	HMDB00848	6426855		1	16.06	0.0006
5-amino-1-(5-phospho-D-ribosyl)imidazole	HMDB01235	161500	C03373	1	19.97	0.0238
1-methylnicotinamide	HMDB00699	457	C02918	1	231.03	0.0057

* n=3 repeats, p value was calculated using two-tailed unpaired Student’s t-test.

Supplementary Table 2 Metabolites that are significantly altered in siHNF4A vs siNeg HepG2 cells

Compound name	HMDB	PubChem	KEGG	Relative abundance		* p value
				HepG2 siNeg	HepG2 siHNF4A	
CMP-N-trimethyl-2-aminoethylphosphonate	HMDB60072	440755	C05674	1	0.03	0.00011
dTTP	HMDB01342	64968	C00459	1	0.03	0.00634
Deoxyinosine	HMDB00071	65058	C05512	1	0.04	0.00182
2-deoxycytidine	HMDB00014	13711	C00881	1	0.23	0.00037
C9H13N5O3(4)	NA	NA	NA	1	0.26	0.04075
3-hydroxyhexadecanoylcarnitine	HMDB13336	53481691		1	0.28	0.01944
L-xylonate/L-lyxonate	HMDB60256	6971043	C05411	1	0.32	0.00362
cytosine	HMDB00630	597	C00380	1	0.32	0.00076
decenoyl carnitine	NA	NA	NA	1	0.32	0.00699
butyryl carnitine	HMDB02013	439829	C02862	1	0.33	0.00699
D-Glucuronate 1-phosphate	HMDB03976	440650	C05385	1	0.34	0.00080
Phosphodimethylethanolamine	HMDB60244	NA	C13482	1	0.34	0.00836
24-oxo-1alpha-23-25-trihydroxyvitamin D3	HMDB60129	10478755	NA	1	0.35	0.02249
alpha-linolenyl carnitine/gamma-linolenyl carnitine	HMDB06319	53477821		1	0.36	0.00071
Hypotaurine	HMDB00965	107812	C00519	1	0.38	0.00040
2-aminomuconate	HMDB01241	5280499	C02220	1	0.39	0.00558
decanoyl carnitine	HMDB06210	53477803		1	0.39	0.00030
pseudoecgonine/ecgonine	HMDB06348	443845	C12449	1	0.39	0.00003
N-acetyl-L-aspartate/2-Amino-3-oxoadipate	HMDB00812	65065	C01042	1	0.39	0.00283
tetradecenoyl carnitine	HMDB13329	53481677		1	0.42	0.03306
octanoyl carnitine/L-Octanoylcarnitine	HMDB00791	11953814	C02838	1	0.43	0.00038
lauroyl carnitine	HMDB02250	168381		1	0.43	0.00075
3-Hydroxybutyrylcarnitine	HMDB13127	53481617Ê	NA	1	0.44	0.00059
N-phosphocreatinate	HMDB01511	587	C02305	1	0.44	0.00099
(R)-carnitine	HMDB00062	2724480	C15025	1	0.45	0.00260
octenoyl carnitine	NA	NA	NA	1	0.46	0.00120
UDP-D-glucuronate	HMDB00935	17473	C00167	1	0.46	0.00109
O-Phospho-L-serine	HMDB01721	106	C01005	1	0.47	0.03803
N-Methylethanolamine phosphate	NA	NA	C01210	1	0.47	0.00025
tiglyl carnitine	HMDB02366	22833596	NA	1	0.47	0.01486
3-dehydro-L-gulonate/D-glucuronate/L-iduronate	HMDB06334	439273	C00618	1	0.50	0.00355
O-acetylcarnitine	HMDB00201	1	C02571	1	0.50	0.00105
3-hydroxy-isovaleryl carnitine	NA	NA	NA	1	0.50	0.00350
acetylcholine/4-(trimethylammonio)butanoate	HMDB00895	187	C01996	1	0.53	0.01565
11'-Carboxy-alpha-chromanol	HMDB12515	53481451		1	0.54	0.01205
Sedoheptulose 7-phosphate	METPA0585	NA	C05382	1	0.56	0.02578
4,6-Dihydroxyquinoline/quinoline-4,8-diol	HMDB04077	440738	C05639	1	0.56	0.00110
Cystathionine	HMDB00099	439258	C02291	1	0.59	0.03189
choline phosphate	HMDB01565	1014	C00588	1	0.59	0.00534
trans-Hexadec-2-enoyl carnitine	HMDB06317	53477817		1	0.60	0.00229
Taurine	HMDB00251	1123	C00245	1	0.60	0.00870
gulonate	HMDB03290	152304	C00800	1	0.60	0.00100
Linoleyl carnitine/Linoelaidyl carnitine	HMDB06469	6450015		1	0.60	0.00034
L-threonate	HMDB00943	151152	C01620	1	0.62	0.00221
C16H26O3(4)	NA	NA	NA	1	0.64	0.01419
3-Deoxy-D-glycero-D-galacto-2-nonulosonic acid	HMDB00425	22833524		1	0.65	0.00000
tetradecanoyl carnitine(myristoyl carnitine)	HMDB05066	53477791		1	0.65	0.01373
cytidine	HMDB00089	6175	C00475	1	1.57	0.01469
citr_L[e]	NA	NA	NA	1	1.60	0.00533
Biotin	HMDB00030	171548	C00120	1	1.68	0.00790
sphingosine/(2S)-1-hydroxy-3-oxooctadecan-2-aminium	HMDB00252	5353955	C00319	1	1.68	0.01128
L-4-hydroxyglutamate semialdehyde	HMDB06556	8224	C05938	1	1.69	0.00040

beta-1-4-mannose-N-acetylglucosamine	HMDB06535	53477853		1	1.71	0.01469
L-methionine	HMDB00696	6137	C00073	1	1.72	0.00160
S-[2-carboxy-1-(1 H-imidazol-4-yl)ethyl]-L-cysteine	NA	NA	NA	1	1.72	0.01737
N-acetyl-seryl-aspartate	NA	NA	NA	1	1.78	0.00325
L-histidine	HMDB00177	6274	C00135	1	1.85	0.01143
N(2)-acetyl-L-ornithine	HMDB03357	439232	C00437	1	1.92	0.00869
Dihydroxyacetone phosphate	HMDB01473	668	C00111	1	2.16	0.00119
Glycerol 3-phosphate	HMDB00126	439162	C00093	1	2.25	0.00002
N-acetyl-L-glutamate	HMDB01138	185	C00624	1	2.30	0.00302
3-Hydroxy-N6N6N6-trimethyl-L-lysine	HMDB01422	439460	C01259	1	2.40	0.00082
Glycylproline/L-Prolinylglycine	HMDB00721	79101	NA	1	2.53	0.00626
L-cysteine	HMDB00574	5862	C00097	1	2.71	0.00416
dUMP	HMDB01409	65063	C00365	1	3.85	0.00001

* n=3 repeats, p value was calculated using two-tailed unpaired Student’s t-test.

Supplementary Table 3 Metabolites that are significantly altered in both siHNF4A HepG2 and SNU449 cells

Compound name	HMDB	PubChem	KEGG	Relative abundance		
				HepG2 siNeg	HepG2 siHNF4A	SNU449 siNeg
CMP-N-trimethyl-2-aminoethylphosphonate	HMDB60072	440755	C05674	1	0.03	0.61
Deoxyinosine	HMDB00071	65058	C05512	1	0.04	0.19
C9H13N5O3(4)	NA	NA	NA	1	0.26	0.01
L-xylonate/L-lyxonate	HMDB60256	6971043	C05411	1	0.32	0.27
decenoyl carnitine	NA	NA	NA	1	0.32	0.17
D-Glucuronate 1-phosphate	HMDB03976	440650	C05385	1	0.34	0.23
alpha-linolenyl carnitine/gamma-linolenyl carnitine	HMDB06319	53477821		1	0.36	3.35
Hypotaurine	HMDB00965	107812	C00519	1	0.38	0.29
decanoyl carnitine	HMDB06210	53477803		1	0.39	0.19
pseudoecgonine/ecgonine	HMDB06348	443845	C12449	1	0.39	0.43
octanoyl carnitine/L-Octanoylcarnitine	HMDB00791	11953814	C02838	1	0.43	0.39
lauroyl carnitine	HMDB02250	168381		1	0.43	0.42
3-Hydroxybutyrylcarnitine	HMDB13127	53481617Ê	NA	1	0.44	0.50
N-phosphocreatinate	HMDB01511	587	C02305	1	0.44	0.05
(R)-carnitine	HMDB00062	2724480	C15025	1	0.45	0.40
octenoyl carnitine	NA	NA	NA	1	0.46	0.53
UDP-D-glucuronate	HMDB00935	17473	C00167	1	0.46	0.25
O-Phospho-L-serine	HMDB01721	106	C01005	1	0.47	0.11
N-Methylethanolamine phosphate	NA	NA	C01210	1	0.47	2.59
O-acetylcarnitine	HMDB00201	1	C02571	1	0.50	0.46
acetylcholine/4-(trimethylammonio)butanoate	HMDB00895	187	C01996	1	0.53	0.40
11'-Carboxy-alpha-chromanol	HMDB12515	53481451		1	0.54	0.36
Sedoheptulose 7-phosphate	METPA0585	NA	C05382	1	0.56	0.08
Cystathionine	HMDB00099	439258	C02291	1	0.59	0.20
choline phosphate	HMDB01565	1014	C00588	1	0.59	0.59
trans-Hexadec-2-enoyl carnitine	HMDB06317	53477817		1	0.60	2.92
gulonate	HMDB03290	152304	C00800	1	0.60	0.46
Linoleyl carnitine/Linoelaidyl carnitine	HMDB06469	6450015		1	0.60	6.17
tetradecanoyl carnitine(myristoyl carnitine)	HMDB05066	53477791		1	0.65	2.41
cytidine	HMDB00089	6175	C00475	1	1.57	2.07
citr_L[e]	NA	NA	NA	1	1.60	1.80
Biotin	HMDB00030	171548	C00120	1	1.68	1.86
sphingosine/(2S)-1-hydroxy-3-oxooctadecan-2-aminium	HMDB00252	5353955	C00319	1	1.68	1.54
L-4-hydroxyglutamate semialdehyde	HMDB06556	8224	C05938	1	1.69	1.65
L-methionine	HMDB00696	6137	C00073	1	1.72	1.64
N-acetyl-seryl-aspartate	NA	NA	NA	1	1.78	0.66
L-histidine	HMDB00177	6274	C00135	1	1.85	1.87
N(2)-acetyl-L-ornithine	HMDB03357	439232	C00437	1	1.92	3.25
Glycerol 3-phosphate	HMDB00126	439162	C00093	1	2.25	0.12
N-acetyl-L-glutamate	HMDB01138	185	C00624	1	2.30	2.73
L-cysteine	HMDB00574	5862	C00097	1	2.71	2.95
dUMP	HMDB01409	65063	C00365	1	3.85	0.12

Note: Metabolites highlighted Blue were changed to different directions in HNF4A knockdown HepG2 cells and SNU449 cells.

Supplementary Table 4 MS parameters of LC-MS analysis of SAA pathway metabolites

compound	chemical formula	molecular weight	predicted ion mass	quad isolation	normalized collision energy	extracted ion chromatogram
Methionine	$C_5H_{11}NO_2S$	149.05105	150.058326	150	30	104.05-104.06
MTA	$C_{11}H_{15}N_5O_3S$	297.08956	298.096836	298	40	136.05-126.07
SAM	$C_{15}H_{23}N_6O_5S$	399.14451	399.14451	399	30	250.09-250.1
SAH	$C_{14}H_{20}N_6O_5S$	384.12159	385.128866	385	30	250.07-250.08
Glutathione (red)	$C_{10}H_{17}N_3O_6S$	307.08381	308.091086	308	25	179.04-179.06
Glutathione (ox)	$C_{20}H_{32}N_6O_{12}S_2$	612.15196	613.159236	613	30	355.1-355.2
Cysteine	$C_3H_7NO_2S$	121.01975	122.027026	122	30	76.0-76.05
Homocysteine	$C_4H_9NO_2S$	135.0354	136.042676	136	30	118.0-118.1

Supplementary Table 5 Sequences of primers

Name	Sequence
**qPCR primers**	
18SF	CGTCTGCCCTATCAACTTTCG
18SR	TGCCTTCCTTGGATGTGGTAG
ASNSF	GCAGCTGAAAGAAGCCCAAGT
ASNSR	TGTCTTCCATGCCAATTGCA
ATF4F	CCCTTCACCTTCTTACAACCTC
ATF4R	TGCCCAGCTCTAAACTAAAGGA
BHMTF	TGGAGAACAGGGGCAACTATG
BHMTR	CTGACTCACTCCTCCTGCTAC
CBSF	GGCCAAGTGTGAGTTCTTCAA
CBSR	GGCTCGATAATCGTGTCCCC
CDOF	GGGCAATGTACGCCAAGTTC
CDOR	TGAATACTGCTGCCATGTCCT
CHOPF	GAACGGCTCAAGCAGGAAATC
CHOPR	TTCACCATTCGGTCAATCAGAG
COL3A1F	GGTGTCCCAGGGAAAGATGG
COL3A1R	TATACCTGGAAGTCCGGGGG
COL4A5F	CAACCCCTAAAGGGCCAGAG
COL4A5R	TGTGAACTGCGATCACCACA
COL4A6F	GAATGAAGGGGAAGGCAGGG
COL4A6R	TAGACCCAAGGGTCCAGGAG
COL6A1F	TCAAAAATAACGTGGAGCAAGTGTG
COL6A1R	CCCCGGCTTGCCTCGTT
CPED1F	CTGCCAGTGGATGGAGTACATT
CPED1R	TGAATGTGTCAACTACTTCATAGCC
CTHF	CATGAGTTGGTGAAGCGTCAG
CTHR	AGCTCTCGGCCAGAGTAAATA
EcadherinF	AGTGGGCACAGATGGTGTGA
EcadherinR	TAGGTGGAGTCCCAGGCGTA
Fibronectin1F	CTGAAAGACCAGCAGAGGCA
Fibronectin1R	GTGTAGGGGTCAAAGCACGA
HNF1AF	AACACCTCAACAAGGGCACTC
HNF1AR	CCCCACTTGAAACGGTTCCT
HNF4AF	CGAAGGTCAAGCTATGAGGACA
HNF4AR	ATCTGCGATGCTGGCAATCT
ITGAVF	GACCATCTCATCACTAAGCGGG
ITGAVR	AGCACTGAGCAACTCCACAAC
MAT1AF	ATCAGGGTTTGATGTTCGGCT
MAT1AR	GCGTTGAGCTTGTGAGCAA
MRSF	AGGCCCGGAAGATTGTGTG
MRSR	AGAGGTAGTTGCCGCTATCCA
N-cadherinF	GAACGCCAGGCCAAACAACTT
N-cadherinR	TGCAGCTGGCTCAAGTCATAGT
TWIST1F	GTCCGCAGTCTTACGAGGAG
TWIST1R	GCTTGAGGGTCTGAATCTTGCT
VimentinF	CCTCACCTGTGAAGTGGATGC
VimentinR	CAACGGCAAAGTTCTCTTCCA
**ChIP-PCR primers**	
BHMTF	GGACTTCAGTCTTATGACCTCCC
BHMTR	AGGAAGTGAGTTCTGCAATAAACTG
CBSF	GGCATTGTGTCAGAGGGTGA
CBSR	CACTGCTCCTTAGCAGGCAT
CDO1F	GCTTCAAATCTCCATCCTTTGGTC
CDO1R	CTTGACATTTGTGGTGTTTGAGGT

CTHF	CTAAGCCAGCTTTCCGCCAC
CTHR	CCTGGGTCCAAGTGGTGTTAG
MAT1AF	CGGGGTAAATCTGACTGAAAGAGA
MAT1AR	TTAAGTCCTCCCTTACGCACA
**Luc reportor constructs primers**	
MAT1A	
MAT1A Promoter F	CGAGCTCTTACGCGTGCTAGCTCGAAGCTCCATCACTAAACTGCTC
MAT1A Promoter R	ACTTAGATCGCAGATCTCGAGCACACAGTTGATCTCACTGACATGC
MAT1A Mut F	CTCCAGAGGCTTGTCAGTGAAGCTTTTTTTTTGAAGGCTGCTGCATGTCAGTGAG
MAT1A Mut R	CTCACTGACATGCAGCAGCCTTCAAAAAAAAAGCTTCACTGACAAGCCTCTGGAG
BHMT	
BHMT Promoter F	CGAGCTCTTACGCGTGCTAGCTATGACCTCCCTCATCTGGTTGACT
BHMT Promoter R	ACTTAGATCGCAGATCTCGAGCCAGGTGATCCATCCGCCTCGGCCT
BHMT Mut F	CATCTGGTTGACTCCAGTAGGGGGGGGGGGGGTAGGCAGCCTCCCAAGGACAG
BHMT Mut R	CTGTCCTTGGGAGGCTGCCTACCCCCCCCCCCCCTACTGGAGTCAACCAGATG
CBS	
CBS Promoter F	CGAGCTCTTACGCGTGCTAGCGAGGCCGGAGTGGAAGCAGCGA
CBS Promoter R	ACTTAGATCGCAGATCTCGAGAGCCACAGACCCTCCAAATTCCCATTC
CBS Mut F	TGGAAGCAGCGAGGGTGTGAAGGGGGGGGGGGGGGGGGAAGGCAGAAGCAAGCAGGTG
CBS Mut R	CACCTGCTTGCTTCTGCCTTCCCCCCCCCCCCCCCCCTTCACACCCTCGCTGCTTCCA

Supplementary References

1. Qiu, Z. *et al.* A Pharmacogenomic Landscape in Human Liver Cancers. *Cancer Cell* **36**, 179-193 e111 (2019).
2. Davis, C.A. *et al.* The Encyclopedia of DNA elements (ENCODE): data portal update. *Nucleic Acids Res* **46**, D794-D801 (2018).
