# Supplementary material for: HNF4α regulates sulfur amino acid metabolism and confers sensitivity to methionine restriction in liver cancer
Source: Nat Commun. 2020 Aug 7;11:3978. doi: 10.1038/s41467-020-17818-w (PMC7414133; doi:10.1038/s41467-020-17818-w)
Supplement: Supplementary file 3 — Reporting Summary [file 41467_2020_17818_MOESM3_ESM.pdf]

nature research

Corresponding author(s): Igor Shats, Xiaoling Li

Last updated by author(s): Jul 8, 2020

Reporting Summary

Nature Research wishes to improve the reproducibility of the work that we publish. This form provides structure for consistency and transparency in reporting. For further information on Nature Research policies, see Authors & Referees and the Editorial Policy Checklist .

Statistics

For all statistical analyses, confirm that the following items are present in the figure legend, table legend, main text, or Methods section.

n/a	Confirmed	
☐	☒	The exact sample size (*n*) for each experimental group/condition, given as a discrete number and unit of measurement
☐	☒	A statement on whether measurements were taken from distinct samples or whether the same sample was measured repeatedly
☐	☒	The statistical test(s) used AND whether they are one- or two-sided<br>*Only common tests should be described solely by name; describe more complex techniques in the Methods section.*
☒	☐	A description of all covariates tested
☐	☒	A description of any assumptions or corrections, such as tests of normality and adjustment for multiple comparisons
☐	☒	A full description of the statistical parameters including central tendency (e.g. means) or other basic estimates (e.g. regression coefficient) AND variation (e.g. standard deviation) or associated estimates of uncertainty (e.g. confidence intervals)
☐	☒	For null hypothesis testing, the test statistic (e.g. *F*, *t*, *r*) with confidence intervals, effect sizes, degrees of freedom and *P* value noted<br>*Give P values as exact values whenever suitable.*
☒	☐	For Bayesian analysis, information on the choice of priors and Markov chain Monte Carlo settings
☒	☐	For hierarchical and complex designs, identification of the appropriate level for tests and full reporting of outcomes
☐	☒	Estimates of effect sizes (e.g. Cohen's *d*, Pearson's *r*), indicating how they were calculated

Our web collection on statistics for biologists contains articles on many of the points above.

Software and code

Policy information about availability of computer code

Data collection

The processed TCGA RNA-seq gene expression data for 50 normal and 373 tumor LIHC samples from The Cancer Genome Atlas - Data Portal (https://portal.gdc.cancer.gov/).
Cellular metabolites were profiled using Sieve 2.0 (Thermo Scientific), and individual SAA metabolites were processed using the Qual Browser application in the Xcalibur software suite 4.2.28.14 (Thermo Scientific).
Quantitative real-time PCR data were collected in Bio-rad CFX manager 3.1.
Cell culture images were taken in EVOS FL Auto Cell Imaging System Software, version 1.
Cell migration images were taken in
Immuno-blots and dot-blots were scanned in Li-COR Odyssey software 3.0 on Odyssey infrared imager.

Data analysis

Correlation analysis: we computed the pair-wise Pearson correlation coefficient and the corresponding p-value between two genes using Matlab 2018a.
Survival analysis: our analysis is based on patients' survival from the time of TCGA biospecimen procurement to death or last follow-up. Specifically, the "curated post-procurement survival" is calculated as follows, post-procurement survival = days_to_last_contact – days_to_sample_procurement. If a patient has multiple follow-ups, we used the latest lost-to-follow-up date or the earliest death date. In addition, we filtered out one patient with negative "post-procurement survival". We calculated the coefficient estimate (beta value) and p-value using Cox proportional hazards regression implemented in Matlab, and we visualized the survival distribution with Kaplan-Meier survival curves. Note that the Kaplan-Meier survival curves were generated using samples whose expression levels were among the top and bottom 33% of expression values for corresponding genes.
Clustering: For each gene, we standardized its expression levels across the 371 samples to a mean of 0 and standard deviation of 1. Next, any data points with a standardized value less than the negative of the maximum standardized value (i.e., 5.5) were assigned to -5.5. Only 14 data points out of 8,206 were reassigned. This reassignment was to ensure that the colors in the heatmap were balanced. We then carried out a two-way hierarchical clustering analysis using the Euclidean distance metric for (dis)similarity measure and displayed the clustering results using a heatmap.
Student's t-test for in vitro experiments was performed in Microsoft Excel 16.16.23. Mann-Whitney test for in vivo experiments was performed in Prism 8.

Metabolite analysis was performed in MetaboAnalyst 4.0.
Dot blots and immuno-blots were analyzed in ImageJ 2.0.0 (Fiji).
FACS analysis data were analyzed in BD FACSDiva 8.0.2.
Quantitative real-time PCR data were analyzed in Microsoft Excel 16.16.23.

For manuscripts utilizing custom algorithms or software that are central to the research but not yet described in published literature, software must be made available to editors/reviewers. We strongly encourage code deposition in a community repository (e.g. GitHub). See the Nature Research guidelines for submitting code & software for further information.

Data

Policy information about availability of data

All manuscripts must include a data availability statement. This statement should provide the following information, where applicable:

- Accession codes, unique identifiers, or web links for publicly available datasets
- A list of figures that have associated raw data
- A description of any restrictions on data availability

The TCGA RNA-seq gene expression data were downloaded from The Cancer Genome Atlas - Data Portal (https://portal.gdc.cancer.gov/).
The RNA-seq data of different human liver cancer cell lines were downloaded from Broad Institute Cancer Cell Line Encyclopedia (CCLE, https://portals.broadinstitute.org/ccle/) and from Liver Cancer Model Repository (LIMORE, https://www.picb.ac.cn/limore/) .
Metabolomics data are provided in Supplementary Tables.
The source file for unprocessed and uncompressed immuno-blots used in the Figures is available in supplementary Source file.

Field-specific reporting

Please select the one below that is the best fit for your research. If you are not sure, read the appropriate sections before making your selection.

[x] Life sciences [ ] Behavioural & social sciences [ ] Ecological, evolutionary & environmental sciences

For a reference copy of the document with all sections, see nature.com/documents/nr-reporting-summary-flat.pdf

Life sciences study design

All studies must disclose on these points even when the disclosure is negative.

Sample size	For in vitro cellular experiments, standard sample size n=3-5 was chosen based on empirical experience, not a power calculation.<br>For in vivo animal experiment in Figure 6H and 6I, n=8 was chosen to achieve 80% of statistic power using Student's t-test of tumor size and weight.<br>The in vivo DEN/HFD liver cancer model in Supplementary Figure 8 was performed as a pilot experiment with n=4 mice/group.
Data exclusions	For the pair-wise correlation analysis using TCGA LIHC RNA-seq expression data, outlier samples that fall below Q1 − 3.0 IQR or above Q3 + 3.0 IQR were removed. For HNF4A-MAT1A correlation, 8 samples were removed. For all other correlation analyses with HNF4A, four samples were removed. No samples were excluded in other experiments. These data were excluded as they were outliers based on the interquartile range method, a common method used for outlier detection. We excluded less than 1.5% of the data in the pair-wise correlation analysis. The data exclusion did not change the conclusion for correlation.
Replication	Metabolite profiling was done with three replicates (from three separate wells of cells cultured at the same time, performed at a Duke facility). Individual SAA metabolites were analyzed and confirmed from at least three independent experiments with three repeats each time at the NIEHS Mass Spectrometry facility using different chromatography. All repeats were successful.<br>All other in vitro cellular experiments were performed in at least triplicates and repeated at least three times. All repeats were successful.<br>The in vivo animal experiment in Figure 6H and 6I was performed with 4 mice per group and 2 tumor injections/mouse. Because not all injections resulted in tumor growth, so the final tumor number was 5-7/group.<br>The in vivo DEN/HFD liver cancer model in Supplementary Figure 8 was performed as a pilot experiment with n=4 mice/group. Due to the current COVID-19 pandemic, we were not able to initiate and complete a larger repeat for this experiment.
Randomization	In all in vitro cellular experiments, samples were randomized into different treatment groups.<br>In both in vivo animal experiments, mice were randomized to experimental groups.
Blinding	All experiments were not analyzed blindly, as this study does not address the effect or efficacy of an agent that requires blinding.

Reporting for specific materials, systems and methods

We require information from authors about some types of materials, experimental systems and methods used in many studies. Here, indicate whether each material, system or method listed is relevant to your study. If you are not sure if a list item applies to your research, read the appropriate section before selecting a response.

Materials & experimental systems

n/a	Involved in the study	
	x	Antibodies
	x	Eukaryotic cell lines
x		Palaeontology
	x	Animals and other organisms
x		Human research participants
x		Clinical data

Methods

n/a	Involved in the study	
x		ChIP-seq
	x	Flow cytometry
x		MRI-based neuroimaging

Antibodies

Antibodies used

HNF4a (Santa Cruz, sc-374229, clone H-1, Lot B2417), BHMT (Santa Cruz, sc-390299, clone H-7, Lot B1913), CBS (Santa Cruz, sc-133154, clone B-4, Lot D0918), CDO1 (Thermo Fisher Scientific, PA5-38005, Lot UD2757664A), MAT1A (Abcam, ab129176, GR91375-9), CTH (Proteintech Group, 12217-1-AP, Lot 00076987), Actin (Millipore Sigma, MAB1501, clone C4, Lot 3132961), Puromycin (Millipore Sigma, MABE343, clone 12D10, Lot 3379285), HRP-conjugated alpha Tubulin (Proteintech, HPR-66031, mouse monoclonal, Lot 21000018), HNF4a for CHIP analysis (Abcam, ab41898, clone K9218, CHIP grade, Lot GR4841-65), Ki67 for IHC (Abcam, ab1667, clone SP6, Lot GR3185488-3, 1:150).

Validation

Immuno-blotting assays for HNF4a (mouse, 1:500), BHMT (mouse, 1:500), CBS (mouse, 1:1000), CDO1 (rabbit, 1:1000), MAT1A (rabbit, 1:1000), CTH (rabbit, 1:1000), Actin (mouse, 1:10,000), Puromycin (mouse, 1:1000), HRP-Tubulin (mouse, 1:2000). All these antibodies were validated using siRNAs samples (Figure 4c). HNF4a antibody for CHIP analysis (mouse, 2 ug antibody per 25 ug of DNA) was validated using HNF4A negative SNU449 cells (Figure 4a).

Eukaryotic cell lines

Policy information about cell lines

Cell line source(s)

Huh7, Hep3B, HepG2 and SNU449 were obtained from the Cell Repository at the Tissue Culture Facility of the UNC Lineberger Comprehensive Cancer Center, all of them were originated from ATCC. SNU475 was purchased directly from ATCC.

Authentication

All cell lines were authenticated by the original sources. The techniques/ procedures used to authenticate each cell line:

Mycoplasma contamination

All cell lines were tested negative for the mycoplasma contamination by the Quality Assurance Laboratory of the National Institute of Environmental Health Sciences.

Commonly misidentified lines
(See ICLAC register)

No commonly misidentified lines were used.

Animals and other organisms

Policy information about studies involving animals; ARRIVE guidelines recommended for reporting animal research

Laboratory animals

Mouse: NU/J ((#002019, Jackson Laboratory), female, 6-8 weeks old; C57BL/6 (Shanghai Lingchang Biotechnology), male, 2 weeks old to 12 months old. All mice were housed in a room with a constant temperature (19-23°C) and humidity (55% ± 10%) and a 12-hour light/dark cycle, and had free access to water and food.

Wild animals

The study did not involve wild animals.

Field-collected samples

The study did not involve samples collected from the field.

Ethics oversight

The xenograft experiment was approved by the Institutional Animal Care and Use Committees of the National Institute of Environmental Health Sciences, and the DEN/HFD liver cancer model was approved by Institutional Animal Care and Use Committees of Shanghai Jiao Tong University School of Medicine.

Note that full information on the approval of the study protocol must also be provided in the manuscript.

Flow Cytometry

Plots

Confirm that:

- [x] The axis labels state the marker and fluorochrome used (e.g. CD4-FITC).
- [x] The axis scales are clearly visible. Include numbers along axes only for bottom left plot of group (a 'group' is an analysis of identical markers).
- [x] All plots are contour plots with outliers or pseudocolor plots.
- [x] A numerical value for number of cells or percentage (with statistics) is provided.

Methodology

Sample preparation	siNeg HepG2, siHNF4a HepG2, and siNeg SNU449 cells cultured in CM or MCR medium for 18 hours. For cell ROS analysis, cells were incubated with 500 nM CellRox Green (ThermoFisher Scientific) for 30 min, and immediately analyzed by flow cytometry. For cell cycle analysis, cells were incubated with 10 uM EdU for 1 hr and then prepared for analysis according to manufacturer's instructions of Click-iT EdU Flow Cytometry Assay Kit (ThermoFisher Scientific)
Instrument	BD LSRFortessa
Software	BD FACSDiva 8.0.2.
Cell population abundance	10, 000 single cell events were collected for each sample
Gating strategy	For ROS analysis, debris were excluded using FSC/SSC plot followed by exclusion of cell aggregates using FSC-A/FSC-H plots. MFI of CellRox Green of the resultant single- cell population recorded.<br>For cell cycle analysis, debris and cell aggregates were excluded as described above. Distinct single-cell populations representing G1, S, and G2/M cell cycle phases were defined using rectangular gates on DNA/EdU plots.

- [x] Tick this box to confirm that a figure exemplifying the gating strategy is provided in the Supplementary Information.
