# Supplementary material for: HNF4α regulates sulfur amino acid metabolism and confers sensitivity to methionine restriction in liver cancer
Source: Nat Commun. 2020 Aug 7;11:3978. doi: 10.1038/s41467-020-17818-w (PMC7414133; doi:10.1038/s41467-020-17818-w)

Uncut gels for Fig. 1g

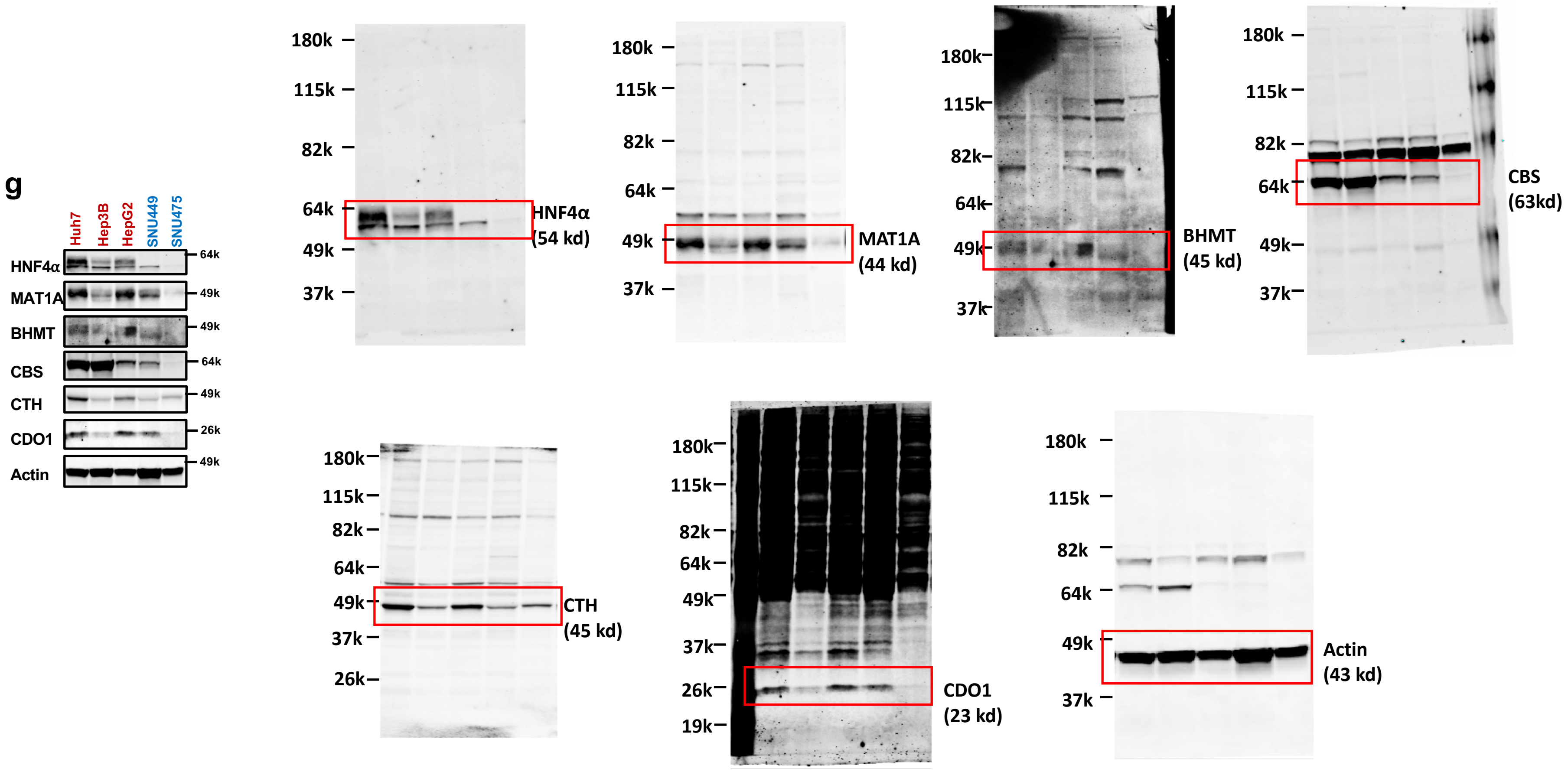

Uncut gels for Fig. 4c

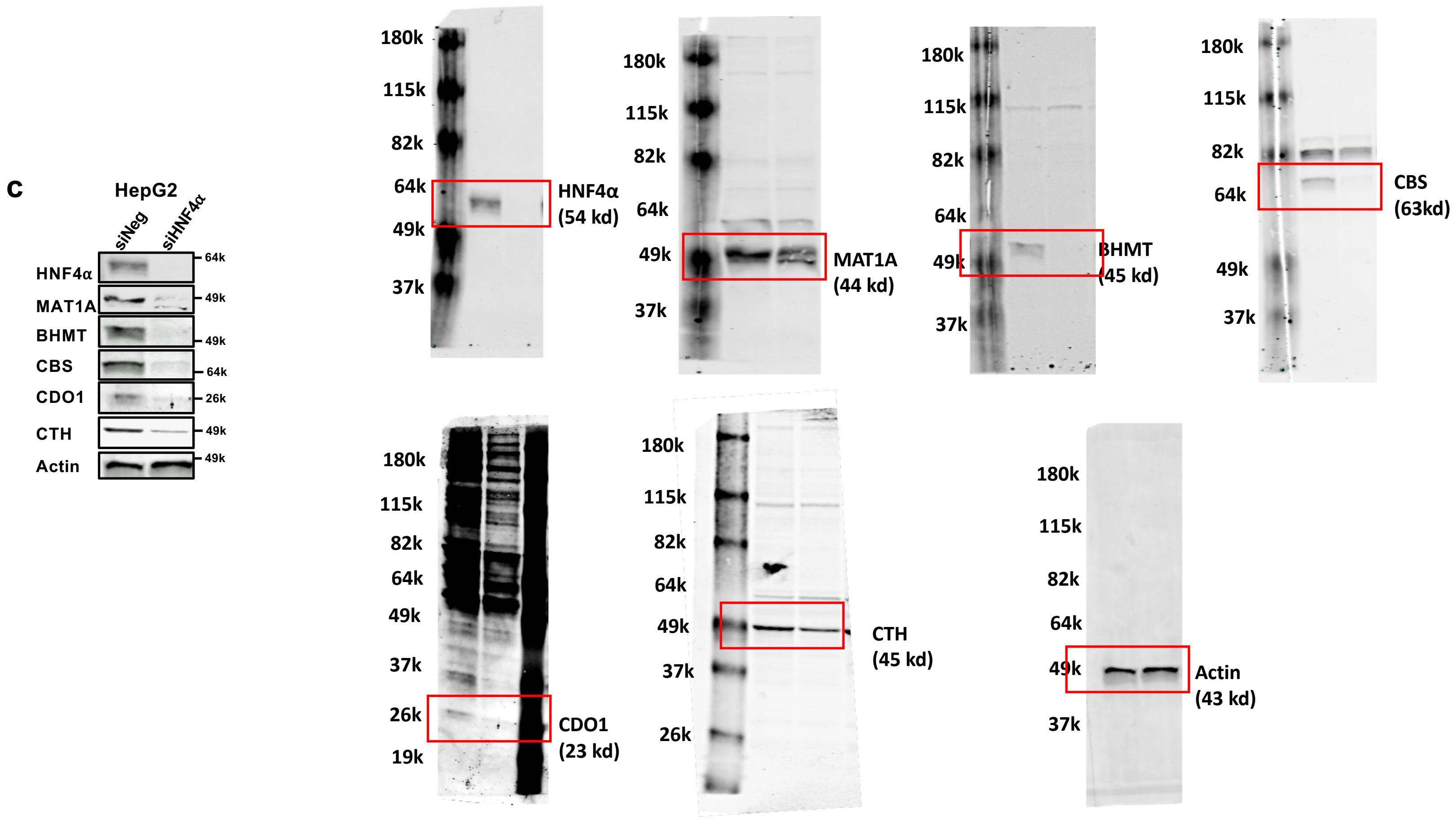

Uncut gels for Fig. 6e

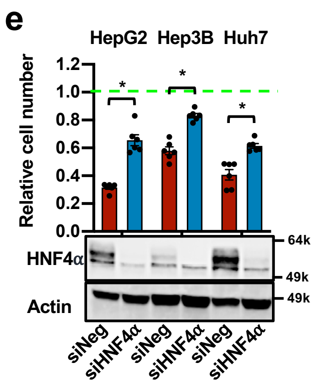

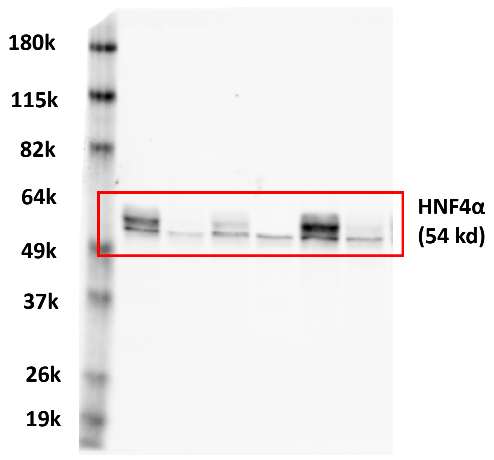

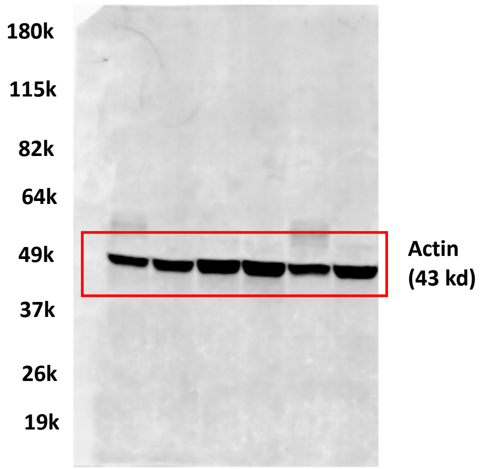

Uncut gels for Supplementary Fig. 4e

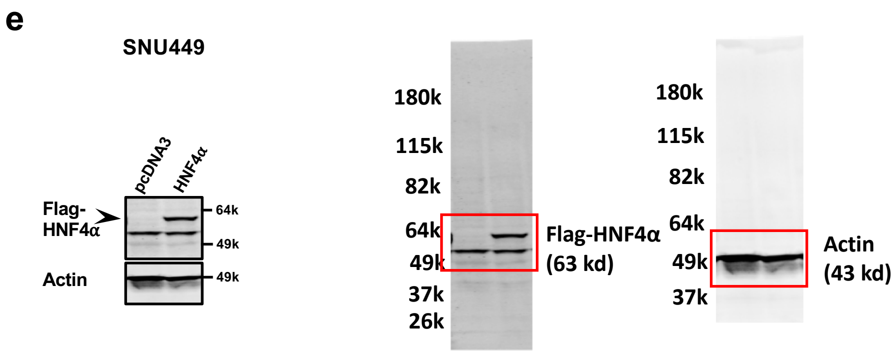

Uncut gels for Supplementary Fig. 6d

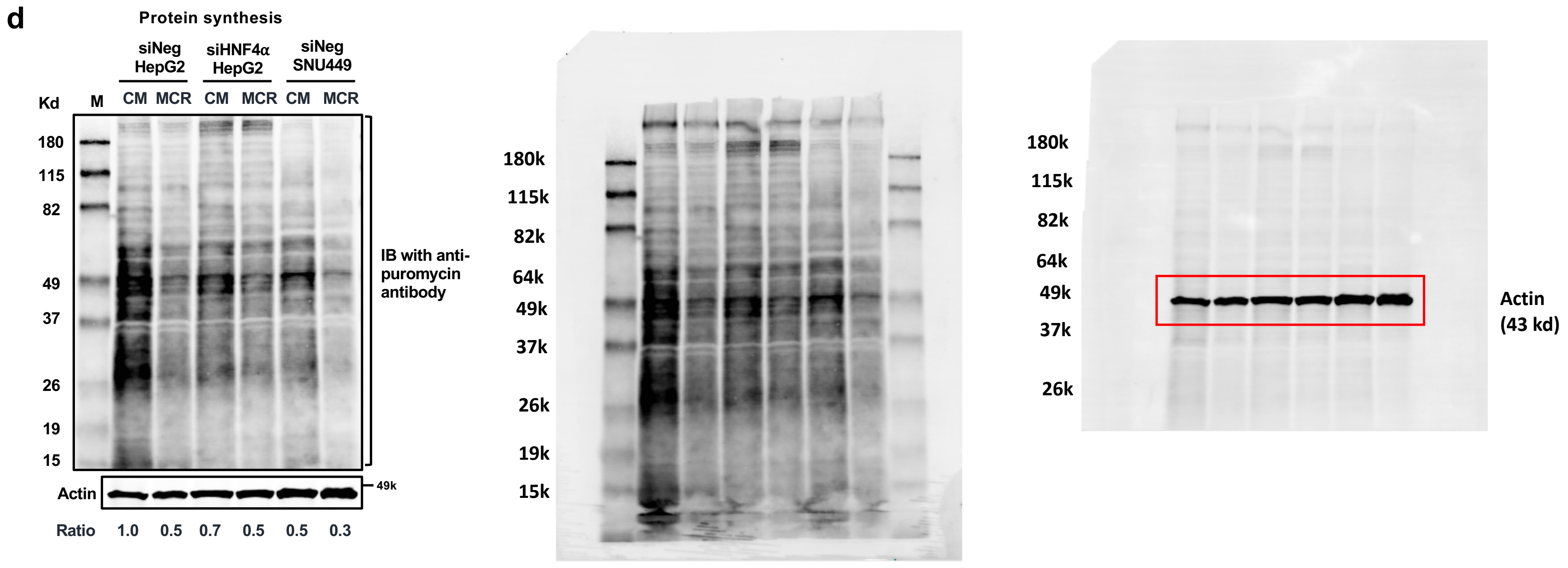

Uncut gels for Supplementary Fig. 8e

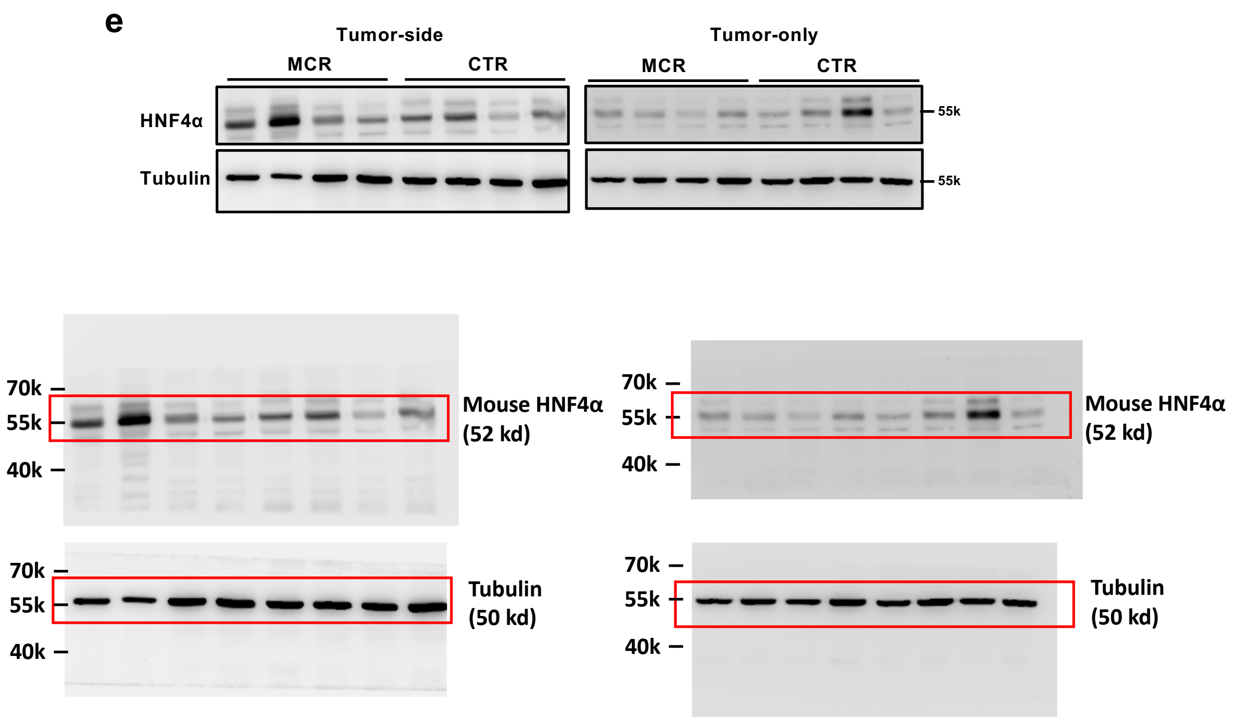

Uncut gels for Supplementary Fig. 10d

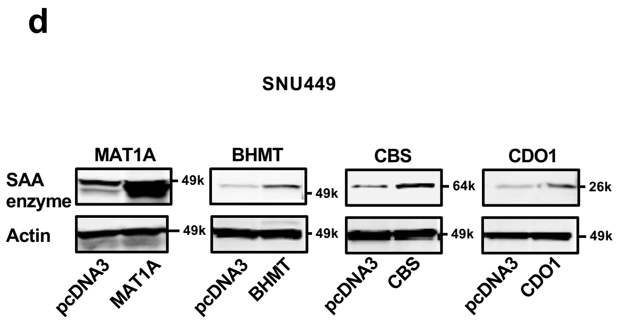

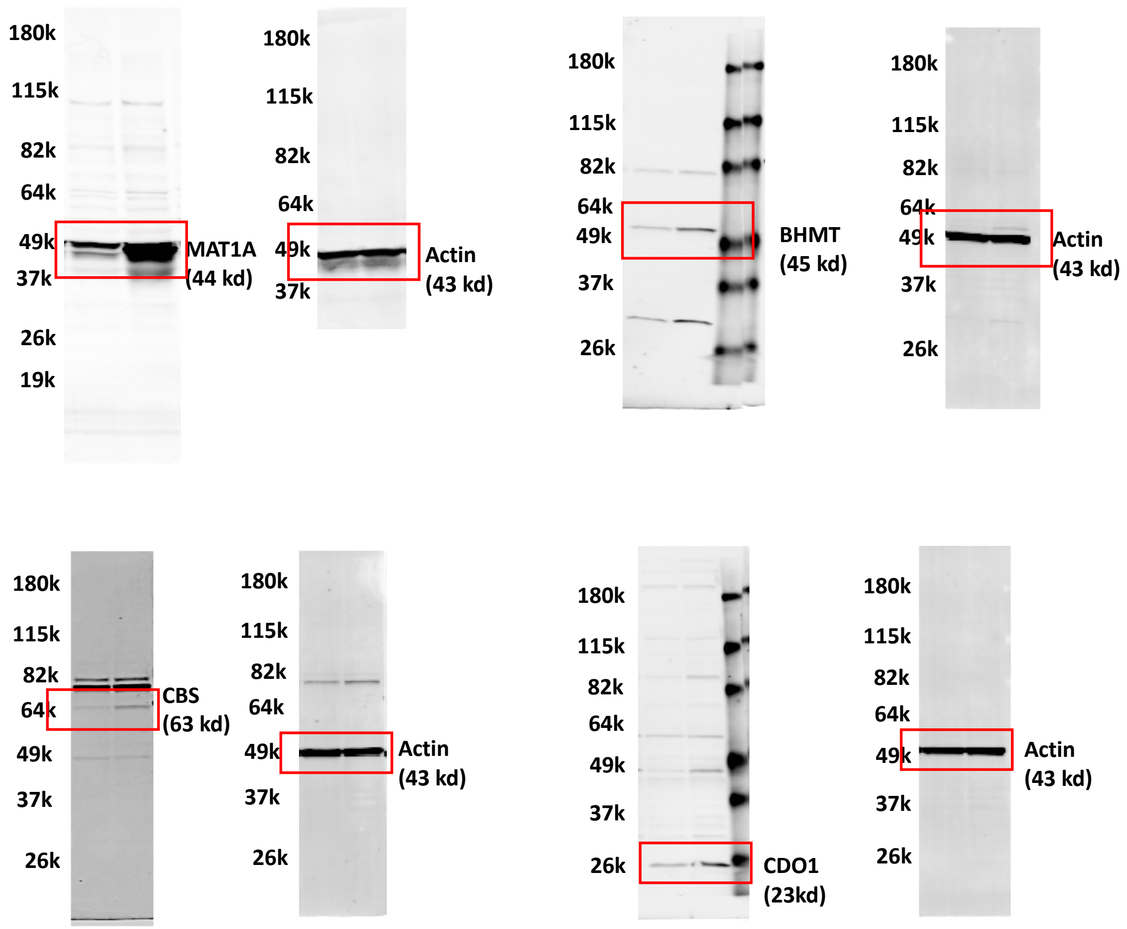

Supplement: Supplementary file 4 — Source Data [file 41467_2020_17818_MOESM4_ESM.pdf]
